# Object detection through search with a foveated visual system

**DOI:** 10.1371/journal.pcbi.1005743

**Published:** 2017-10-09

**Authors:** Emre Akbas, Miguel P. Eckstein

**Affiliations:** 1 Department of Psychological and Brain Sciences, University of California, Santa Barbara, Santa Barbara, California, United States of America; 2 Department of Computer Engineering, Middle East Technical University, Ankara, Turkey; 3 Institute for Collaborative Biotechnologies, University of California, Santa Barbara, Santa Barbara, California, United States of America; Technische Universitat Chemnitz, GERMANY

## Abstract

Humans and many other species sense visual information with varying spatial resolution across the visual field (foveated vision) and deploy eye movements to actively sample regions of interests in scenes. The advantage of such varying resolution architecture is a reduced computational, hence metabolic cost. But what are the performance costs of such processing strategy relative to a scheme that processes the visual field at high spatial resolution? Here we first focus on visual search and combine object detectors from computer vision with a recent model of peripheral pooling regions found at the V1 layer of the human visual system. We develop a foveated object detector that processes the entire scene with varying resolution, uses retino-specific object detection classifiers to guide eye movements, aligns its fovea with regions of interest in the input image and integrates observations across multiple fixations. We compared the foveated object detector against a non-foveated version of the same object detector which processes the entire image at homogeneous high spatial resolution. We evaluated the accuracy of the foveated and non-foveated object detectors identifying 20 different objects classes in scenes from a standard computer vision data set (the PASCAL VOC 2007 dataset). We show that the foveated object detector can approximate the performance of the object detector with homogeneous high spatial resolution processing while bringing significant computational cost savings. Additionally, we assessed the impact of foveation on the computation of bottom-up saliency. An implementation of a simple foveated bottom-up saliency model with eye movements showed agreement in the selection of top salient regions of scenes with those selected by a non-foveated high resolution saliency model. Together, our results might help explain the evolution of foveated visual systems with eye movements as a solution that preserves perceptual performance in visual search while resulting in computational and metabolic savings to the brain.

## Author summary

A large number of species from primates to shrimps do not see the visual world with uniform spatial detail. An area with heightened sensitivity to spatial detail, known as the fovea in mammals is oriented through eye and head movements to scrutinize regions of interest in the visual environment. But why did many species evolve such foveated architecture for vision? Seeing with high spatial detail everywhere requires greater neuronal machinery and energy consumption, thus the advantage of a foveated visual system is to reduce metabolic costs. But does having a foveated visual system incur a price to the performance of the organism in visual tasks? Here, we show using a computer vision object detection model, that the foveated version of the model can attain similar search performance to its non-foveated version that processes the entire visual field with high spatial detail. The results might help explain the evolution of foveated visual systems with eye movements as a solution that preserves perceptual performance while resulting in computational and metabolic savings to the brain.

## Introduction

Many species from primates, birds and shrimps [1, 2] have an area of their visual sensory system with heightened spatial fidelity and utilize eye and head movements to orient this area towards objects of interest in scenes. The pervasiveness of sensory systems with varying spatial resolution for species that heavily rely on vision to sense the world motivates the question about its advantages. The wide-accepted answer is that visual processing with varying spatial resolution reduces the brain’s computational cost. For example, for humans, the density of cones in the fovea is approximately 20 times larger than at 10 degrees into the periphery and 90 times at the far visual periphery [3]. The fovea occupies 0.01% of the retina but utilizes approximately 8-10% of the neuronal machinery in primary visual cortex [4]. A high spatial resolution processing system across the entire visual field matching the fovea’s ratio of primary cortex (V1) neurons per mm of retina would lead to roughly a one thousand increase in the size of the primary visual cortex. A full high spatial resolution visual system would likely drastically increase the brain’s computational expenditures and thus the metabolic cost. The ability of organisms with a heightened area of spatial fidelity (i.e., a fovea) to successfully support perceptual decision making relies critically on the guidance of saccadic eye movements to sample the visual world. Humans perform approximately three eye movements per second. The brain uses peripheral processing to extract critical information and guides the eyes across the scene. Eye movements can be directed to salient regions in a scene as potential locations of interest and for further processing [5–7]. During search, eye movements are also guided by information about the target including basic features including color, size, orientation and shape [8–11], probabilities of object occurrence, global scene statistics [12, 13], object co-occurrence [14–16], and knowledge of the detectability of objects across the visual field [17] to successfully detect objects in cluttered scenes. The brain is also able to acquire information in the visual periphery to guide eye movements concurrent with analyses of information at the foveal region [18]. This foveated visual system with guided eye movements reduces the brain’s computational cost. What is not known are the decision accuracy costs of a foveated architecture relative to a non-foveated high spatial resolution system in ecologically relevant tasks. The current work aims at answering this question.

There have been many proposals for computational models of search and artificial systems with foveated visual systems [19–23]. A number of models use an ideal Bayesian observer that searches for a known target in noise-limited images [17, 24, 25]. Such frameworks typically do not model the degradation in retinal eccentricity with varying resolution feature extraction and the approach is limited to synthetic images for which the statistical distribution of the noise is known. There are other object detectors that can be applied to real world images but use the same high resolution representation of the target (template) across the whole visual field [26, 27]. One group has implemented in hardware a visual sensing approach with two components: a pre-attentive component providing a fixed field of view (FOV) at low resolution, and a localized shiftable FOV at high resolution, designed to recognize and interpret events detected by the pre-attentive system [19, 20]. However, no previous study has implemented a physiologically based foveated visual system and compared its performance for ecologically relevant search tasks against a homogeneous high resolution (non-foveated) system to specifically assess the decision accuracy costs of incorporating a varying resolution system.

The goal of the present work is to investigate the impact on object search performance of using a foveated visual field with physiologically plausible cortical peripheral pooling and saccade exploration, and compare it to a visual system with access to high spatial resolution at all points in the scene [28–30]. We evaluate the accuracy of the models in finding different objects in real scenes. To allow for a fair evaluation of the performance costs of foveation, both models (foveated and non-foveated) need to be implemented within a common computational framework.

Our reasoning is that if a foveated object detection model with eye movements can achieve similar object detection accuracy as a non-foveated approach, it might suggest a possible reason for the evolution of foveated systems in organisms: achieving successful object detection while minimizing computational and metabolic costs.

**Fig 1:**
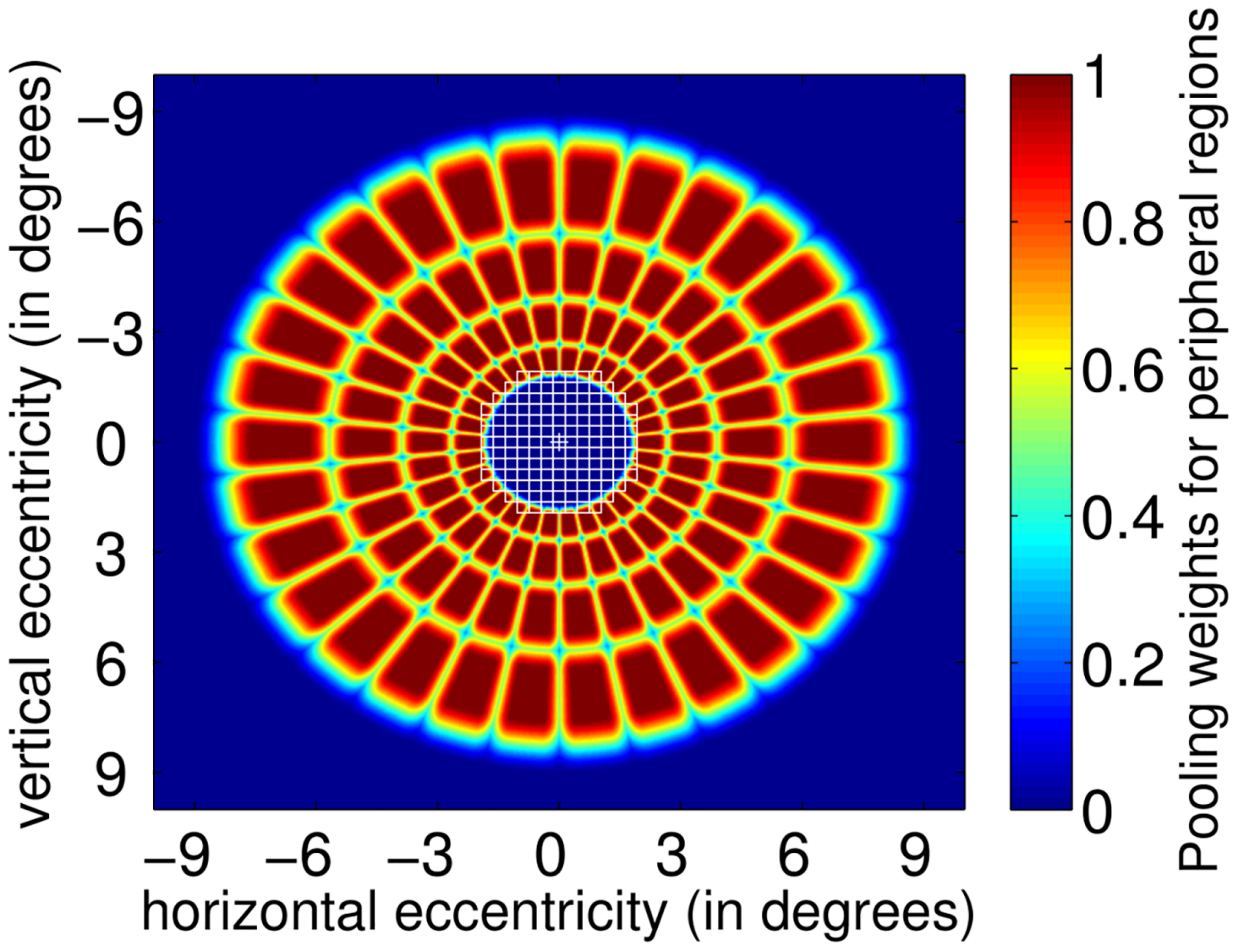
The foveated visual field of the proposed object detector. Square blue boxes with white borders at the center are foveal pooling regions. Around them are peripheral pooling regions which are radially elongated. The sizes of peripheral regions increase with distance to the fixation point which is at the center of the fovea. The color within the peripheral regions represent pooling weights.

To achieve our goal we incorporate a visual field with varying spatial resolution [31–34] to contemporary object detection methods extended to use a latent linear discriminant formulation (§“The foveated object detector (FOD)”). There are many possible methods to implement a foveated visual field in an object detection system. In primates, foveation arises from various properties of the visual system including varying photoreceptor density across the retina [35], larger convergence onto ganglion cells with increasing retinal eccentricity [36, 37], the higher number of neurons per mm^2^ of retina at the visual cortex for foveal areas and spatial pooling possibly contributing to crowding effects (for a review of contributions, see Rosenholtz [38]). In this work, we opt to use a recent model [39] which specifies how responses of elementary sensors are pooled at the layers (V1 and V2) of the human visual cortex. The model specifies the shapes and sizes of V1, V2 regions which pool responses from the visual field. This is clearly a simplification of the multi-stage processing in the human visual system (lower photoreceptor density and higher input convergence at the ganglion cells, lower number of V1 neurons with increasing eccentricity) accounting for the foveated properties of vision. However, such a simplified model seems to capture many aspects of peripheral processing including some crowding effects [38, 39]. We use a simplified version (only the V1 layer, see §“Foveated visual field” for details) of this model as the foveated visual field of our object detector (Fig 1). We call our detector the foveated object detector (FOD). The FOD computational savings arise from the fewer computations (dot products) related to the coarser spatial sampling of features (due to spatial pooling) in the visual periphery.

Importantly, we used the same computer vision object detection framework to develop a foveated system, and a non-foveated system with homogeneous high spatial resolution across the visual field. The high spatial resolution system is the default object detection model which processes all information in the image with the same feature extraction stages and is known as a sliding window method (SW) in computer vision. The term sliding window refers to a re-current application of the feature extraction and classifier across the entire image by shifting spatial windows defining a region of interest.

We compared performances of the models on a standard computer vision image dataset (PASCAL VOC [40]) allowing for direct evaluation of the impact of a biologically based foveated visual system on the default non-foveated object detector across 20 different classes of objects and about 5000 test images.

## Results

### Overview of the non-foveated object detector

**Fig 2:**
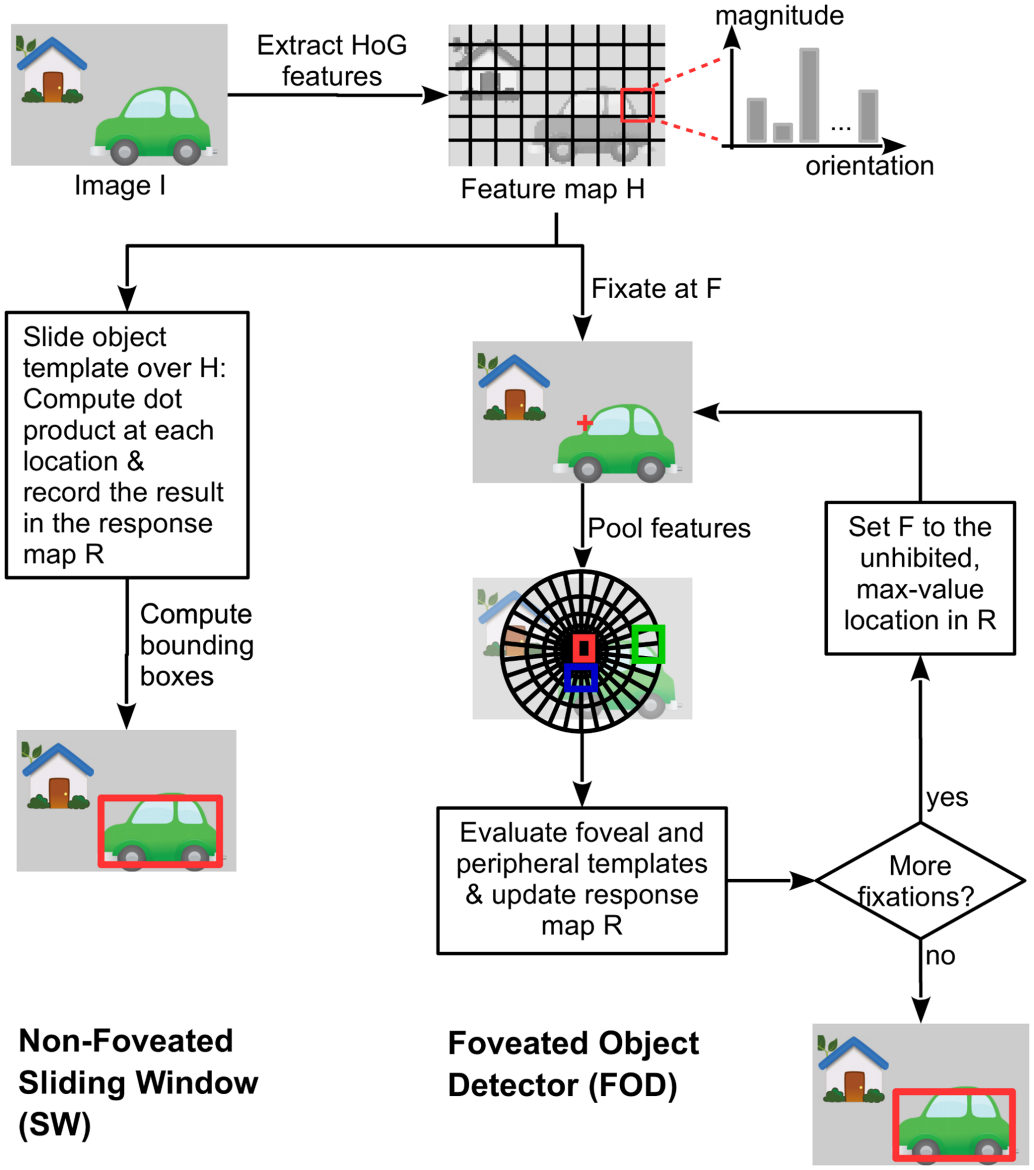
Flowchart of the non-foveated sliding window (SW) model and the foveated object detector (FOD). The feature extraction step is common to both models. First, the image is filtered with simple edge detection filters with different orientations, and gradient magnitude and orientation are estimated at each pixel. Then, the image is divided into small square boxes on a regular grid. Within each box, total gradient magnitude per orientation is computed, which results in a histogram. The output is a collection of feature maps for x, y locations and orientations. For simplicity, only one feature map (H) is shown as input to both models. **Right side**: Foveated Object Detector. The FOD has an initial fixation position that determines the pooling regions of the underlying histogram of gradient features. FOD’s templates are learned through training and are specific to each retinotopic location. The scores reflecting probability of target presence are used to guide saccades to the most likely target location. The object probability scores for each location are integrated across saccades and used for the final perceptual decision.

**Fig 3:**
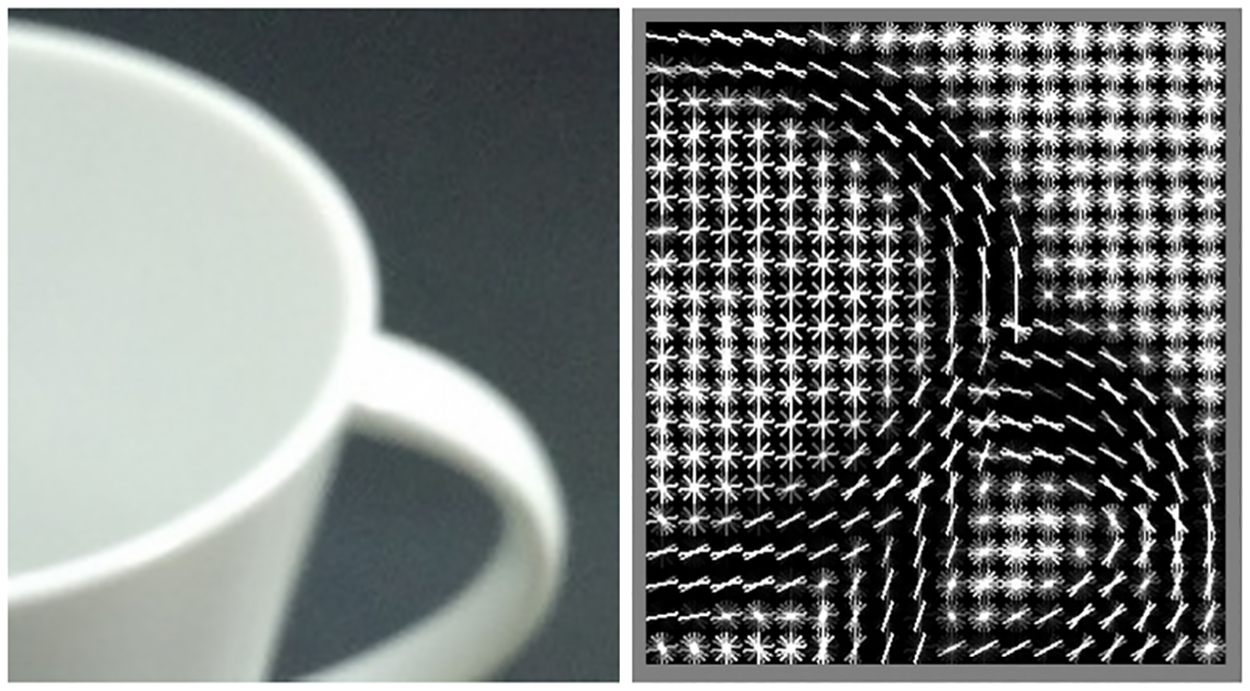
Histogram of oriented gradients (HoG) of a sample image. Left: input image, right: HoG result. First, the input image is convolved with two 1-D filters, namely [+ 1 0 −1] and its transpose. The gradient magnitude and orientation at each pixel are estimated from the convolution results. Then, the image is divided into small, square bins. In each bin, an orientation histogram is computed, which shows the (relative) total gradient magnitude per orientation. Finally, the histogram in each bin is normalized by the total “energy” (e.g. *ℓ*_2_ norm) of a 2x2 block containing the bin akin to divisive local contrast normalization. This final step is known as block normalization. On the right, each HoG bin is represented with short, oriented line segments where brightness encodes the magnitude of the associated orientation. Due to the block normalization, in homogeneous areas (e.g. top-right) all orientations have high and similar magnitudes. (Image source statement: the original picture on the left was taken by the first author.)

The non-foveated object detector, or the sliding window (SW) object detector (Fig 2), starts by extracting from the input image a set of image features known as the histogram of oriented gradients (HoG) [28, 41] (Fig 3) which is a feature descriptor utilized in object detection models in computer vision. The HoGs refer to a distribution of orientations within small neighborhoods akin to the responses of various cell receptive fields with different orientations. The first stage is to convolve the image with a 1-D point discrete derivative kernel (i.e., [-1, 0, 1]) in both of the horizontal and vertical directions. The second stage entails computing the cell histograms. Each pixel within the cell codes a linear response to the various oriented kernels (filters). The local histograms are normalized relative to the mean of a block (Fig 3). This process results in a *M*-by-*N*-by-*D* feature map where *M* is the number of rows, *N* is the number columns and *D* is the number of features which is determined by the number of different edge orientations considered. Next, the feature map is convolved with the object template which was learned from the training images. The object template is a model of object appearance in the form of a *P*-by-*K*-by-*D* matrix of feature weights (typically *P* << *M* and *K* << *N*). The template is evaluated at all *M* * *N* locations on the feature map. Each evaluation is a dot product between the template weights and the HoG features of the spatial *P*-by-*K* region (which corresponds to a bounding box on the image) that is covered by the template. The dot product result is recorded as the detection score for the corresponding bounding box.

The feature extraction and template evaluation process described above considers only a single template and a single scale of the input image, to simplify the explanation. However, in practice, there is uncertainty about the view-point and scale of the object appearing in the scene. Thus, the object detector had more than one template per object class, and each of these templates serves as an appearance model for a distinct view-point of the object (e.g. a bicycle viewed from the front and from the side). In all our experiments, we used two view-point templates per object class. In addition, since the scale of the object sought is not known apriori, a multiscale processing (we used 40 different scales as done in the Deformable Parts Model (DPM) model [28]) of the input image is required. Finally, all bounding boxes with detection scores higher than a given threshold are identified as object detections. However, if there are two bounding boxes that significantly overlap with each other, the one with the lower score is discarded (non-maxima suppression).

### Overview of the foveated object detector (FOD)

#### Feature extraction

The FOD (Fig 2) mimics the process by which humans search for objects in scenes utilizing eye movements to point the high resolution fovea to points of interest. The FOD gets assigned an initial fixation point on the input image and collects information by extracting image features through its foveated visual field. To allow for a fair comparison of models, we equated the feature extraction of the foveated model to that of the non-foveated. We used the histogram of oriented gradients (HoG [28, 41]) as image features and a simplified version of the V1 model [39] to compute pooled features within the visual field. The HoG features are extracted at full resolution over the whole image, however, after V1 pooling, the features around the fixation point are at fine spatial scale while features away from the fixation location are at coarser scale. This fine-to-coarse transition is dictated by the pooling region sizes of the visual field (Fig 1). Furthermore, because of the spatial pooling, a given region of interest has fewer features associated to it as the retinal eccentricity increases. Training such an object detector entails learning templates at all locations in the visual field. We refer to each of these templates as a retino-specific classifer. Because the visual field has varying resolution, the target related features vary depending on where it is located within the visual field. A mixture of linear templates is trained at selected locations in the visual field using a latent-support vector machine-like [28, 42] framework. The section “Methods and models” specifies in detail all aspects of the model and its training. There were a total of around 500-700 different retino-specific classifiers trained to span the entire visual field. Each retino-specific classifier resulted in a object detection score reflecting the strength of evidence for the presence of the searched object at that location.

#### Eye movement strategies

We assessed performance for two eye movement strategies, the maximum-a-posteriori (MAP) rule (§Eye movement strategy) and a random strategy (RAND) to demonstrate the importance of the guidance of eye movements. The MAP eye movement strategy moves the fovea to the location in the image with the highest posterior probability for the presence of the searched target. The MAP model has been shown to be consistent with human eye movements in a variety of visual search tasks [43, 44]. Studies have demonstrated that in some circumstances human saccade statistics better match an ideal searcher [17] that makes eye movements to locations that maximize the accuracy of localizing targets, yet in many circumstances the MAP model approximates the ideal searcher [24, 45] but is computationally more tractable for objects in real scenes.

New object detector scores are generated for each new fixation point. For each fixation, the FOD collects evidence through its foveal and peripheral detection templates and integrates the new evidence into an internal map, which keeps the evidence for target presence at all possible bounding box locations. Briefly, for a certain bounding box location, different fixations yield different detection scores arising from different retino-specific classifiers. The final detection score for that location is the summation of scores obtained through all fixations. The final scores are converted to posterior probabilities using a sigmoid transformation (see §Integrating observations across multiple fixations). The posterior probabilities are utilized to program the next eye movement using the MAP algorithm.

Object detector scores at fixated locations are reduced (inhibition of return) so that the foveated object detector is encouraged explore new locations and avoid revisits (see Part F in S1 Text for details and limitations on implementation of inhibition of return.)

#### Perceptual decision

After multiple eye movements, the FOD integrates, for each spatial location, information collected at different fixations and computes object detection scores and associated bounding boxes. All bounding boxes with detection scores higher than a detection threshold are identified as final object detections. However, if there are two bounding boxes that significantly overlap (intersection over union greater than 0.5) with each other, the one with the lower score is discarded. Known as “non-maxima suppression,” this is a common post-processing step in computer vision object detection.

### Evaluation of the effects of foveation on visual search for objects

We compared two models on the PASCAL VOC 2007 detection (comp3) challenge dataset and protocol [40]. The dataset contains 20 object classes, 5011 training images and 4952 test images. A training image might contain more than one instance of a specific object class. All results are obtained by training the classifiers on a different set of images than those utilized for testing.

#### Measures of performance

For a given object class, the performance of an object detector is computed as follows. First, the object detector is run over the testing images and generates a score for each evaluated location representing the evidence for the object being present at that location. Associated with each score is also a bounding box which encompasses the area of the image associated with the score. Scores are compared to a specific detection threshold *T*. The bounding boxes with scores above the threshold *T* are considered the object detector’s prediction about the presence and location of the objects. To evaluate whether the bounding boxes are considered correct, they are compared against the ground truth bounding boxes surrounding the actual objects in the images. The ground truth is obtained by annotation by multiple humans. A predicted box *P* is deemed a “true positive” if there is a ground truth box *G* such that the intersection area of *P* and *G* divided by the union area of *P* and *G* is larger than 0.5. Otherwise, *P* is deemed a “false positive.”

Next, **recall** and **precision** are computed for the specific threshold *T*. Recall is the hit rate, the number of true positives divided by the number of all ground truth boxes in the testing set. Precision is the number of true positives divided by the number of all predicted boxes retrieved by the detector (the sum of true positives and false positives). By varying the value of *T*, we obtain a recall-precision curve plot (an example is provided in Part A in S1 Text). The area under this curve is called “average precision”, or AP for short. Recall, precision and AP are the most common performance measures utilized in the computer vision community for the object detection problem. The AP can take values in the range [0, 1], however, to show more precision we use the “percent AP” (which is 100 times the original AP score) throughout the paper. To report the performance over many object classes, we average their AP scores which yields the “mean average precision” or “mAP” for short.

#### Comparison of the FOD with non-foveated SW

As a first control, we compared the performance of our non-foveated (SW) implementation which corresponds to only using high-resolution foveal templates only, to three other object detection methods (DPM [28], Examplar-SVM [29], LDA-based detection [42]) which also use sliding window for search, and whose image features (HoG [28, 41]) and recognition models (mixture of linear templates) are similar to ours. We observed that our SW implementation is performing on par with the compared methods. This suggests that the main result in our paper (the influence of foveation on object detector performance) cannot be attributed to the implementation of a low-performing high resolution sliding window approach (SW). The reader is referred to the supplementary section Part B in S1 Text for details of the results of these comparisons.

We compared the performance of the foveated version of our object detector (FOD) with its non-foveated (SW) version. We also evaluated the importance of the eye movement strategy for the FOD by comparing the model with random eye movements vs. the inclusion of the MAP algorithm. Table 1 shows the percent average precision (AP) scores for FOD with different eye movement strategies and different number of fixations. The table also presents the performance of the non-foveated model (SW). The maximum-a-posteriori and random eye movement strategies are denoted with MAP and RAND, respectively. Because the model accuracy results will depend on the initial point of fixation, we ran the models with different initial points of fixation. The presence of a suffix on a model refers to the location of the initial fixation: “-C” stands for the center of the input image, i.e. (0.5, 0.5) in normalized image coordinates where the top-left corner is taken as (0, 0) and the bottom-right corner is (1, 1); and “-E” for the two locations at the left and right edges of the image, 10% of the image width away from the image border, that is (0.1, 0.5) and (0.9, 0.5). MAP-E and RAND-E results are the performance average of two different versions of the foveated models with initial fixations: one with initial fixation close to the left edge of the image, the other run close to the right edge of the image. For the random eye movement, we report the 95% confidence interval for AP over 10 different runs. We ran all systems for a total of 5 fixations. Table 1 shows results for after 1, 3 and 5 fixations. A condition with one fixation is a model that makes decisions based only on the initial fixation. A model with 3 fixations, executes two eye movements, integrates information across the initial fixation and two additional fixations to make a decision about locations of the searched object. The results show that the FOD using the MAP rule with 5 fixations (“MAP-C,5” for short) performs nearly as good as the SW detector (a difference of 0.2% in mean AP).

**Table 1:** Per class percent average precision (AP), mean average precision (mAP) over all 20 classes and relative computational costs of non-foveated SW and FOD on the PASCAL VOC 2007 dataset. (Object class abbreviations are as follows. ap: aeroplane, bk: bike, bd: bird, bt:boat, bl: bottle, bs: bus, cr: car, ct: cat, ch: chair, cw: cow, dt: dining-table, dg: dog, hs: horse, mb: motorbike, pr: person, pt: potted-plant, sh: sheep, sf: sofa, tr: train, tv: tv-monitor).

		ap	bk	bd	bt	bl	bs	cr	ct	ch	cw	dt	dg	hs	mb	pr	pt	sh	sf	tr	tv	mAP	Cost
**SW**		17.5	28.6	9.7	10.4	17.3	29.8	36.7	7.9	11.2	21.0	2.3	2.7	30.9	21.1	19.7	3.0	9.2	13.7	23.5	25.2	17.1	100
**MAP-C**	**1**	17.0	21.1	4.9	9.8	9.3	27.4	27.9	8.5	3.7	12.8	2.0	4.3	29.7	19.7	18.2	1.2	10.7	14.0	26.2	21.8	14.5	11.5
	**3**	17.4	27.7	10.1	10.6	10.4	30.8	31.6	8.4	10.4	17.2	2.1	3.4	33.3	21.1	18.7	3.4	7.6	15.4	26.4	23.5	16.5	31.2
	**5**	17.0	28.6	10.0	10.7	11.2	31.0	34.0	8.3	10.6	18.2	2.1	3.4	34.2	21.8	19.7	2.8	8.1	15.1	27.8	24.0	16.9	49.6
**MAP-E**	**1**	1.6	7.1	4.1	5.6	9.1	8.7	11.7	6.0	3.6	10.2	2.0	2.2	8.5	10.2	13.5	1.3	6.8	8.0	10.6	10.3	7.1	8.7
	**3**	13.0	24.6	9.9	9.8	10.7	27.2	29.3	7.4	10.4	16.4	3.7	2.2	30.6	20.8	16.9	3.3	11.2	13.8	23.0	24.1	15.4	28.1
	**5**	15.1	28.0	9.9	10.4	11.6	29.9	33.0	8.3	10.6	18.7	2.7	4.1	33.7	22.6	18.9	3.1	7.1	14.7	25.5	25.2	16.7	46.9
**RAND**	**1**	8.2	9.3	5.5	9.3	7.8	12.2	16.2	6.1	6.8	7.5	1.6	2.5	10.6	9.1	9.9	1.9	5.0	6.7	11.2	10.0	7.9±1.4	similar to above
	**3**	9.6	13.0	3.2	9.6	9.3	16.9	23.5	8.8	9.4	9.9	1.8	3.2	16.5	12.3	12.2	2.7	3.9	9.3	16.9	11.7	10.2±0.9	
	**5**	10.9	15.3	3.8	9.7	9.6	20.5	26.3	9.3	9.5	10.6	1.5	3.1	20.9	13.7	13.5	2.7	3.9	12.0	18.9	12.4	11.4±1.0	
**RAND-C**	**1**	This row is the same with the “MAP-C, 1” above.																					”
	**3**	17.5	20.4	3.7	10.0	9.3	28.6	27.4	11.5	6.7	11.8	1.7	3.5	31.7	18.0	15.4	2.7	5.4	15.2	26.1	15.8	14.1±0.5	
	**5**	17.6	21.4	5.2	9.9	9.7	28.1	28.6	11.4	9.6	12.1	1.6	3.5	30.0	17.9	15.3	3.7	6.7	14.4	25.4	15.9	14.4±0.7	
**RAND-E**	**1**	This row is the same with the “MAP-E, 1” above.																					”
	**3**	9.1	13.1	2.8	9.7	9.4	17.8	22.5	9.0	6.6	10.7	2.3	3.7	14.9	12.0	14.9	1.3	3.9	2.4	13.6	14.1	9.7±0.7	
	**5**	10.7	15.9	4.1	8.7	9.5	21.9	26.0	8.2	9.7	11.6	1.7	4.3	17.6	13.7	14.1	1.9	5.7	4.8	15.7	15.8	11.1±1.1	

**Fig 4:**
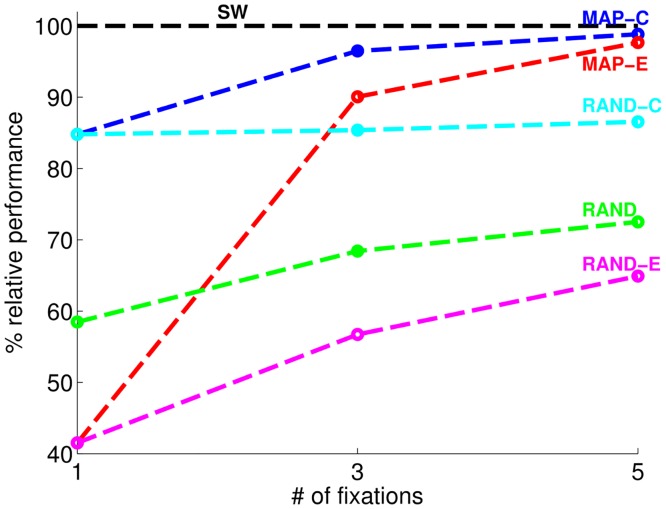
Ratio of mean average precision (AP) scores of FOD systems relative to that of the non-foveated SW system. Graph shows two eye movement algorithms: maximum aposteriori probability (MAP) and random (RAND) and two starting points (C: center of the image; E: left or right edge of the image).

Fig 4 shows the ratio of mean AP for the FOD with the various eye movement strategies to that of the non-foveated SW system (relative performance) as a function of fixation. The relative performance of the MAP-C to non-foveated SW (AP of MAP-C divided by AP of SW) is 98.8% for 5 fixations, 96.5% for 3 fixations and 84.8% for 1 fixation. The FOD with eye movement guidance towards the target (MAP-C,5) achieves or exceeds SW’s performance with only 1 fixation in 4 classes, with 3 fixations in 7 classes, with 5 fixations in 2 classes. For the remaining of 7 classes, FOD needs more than 5 fixations to achieve SW’s performance.

**Fig 5:**
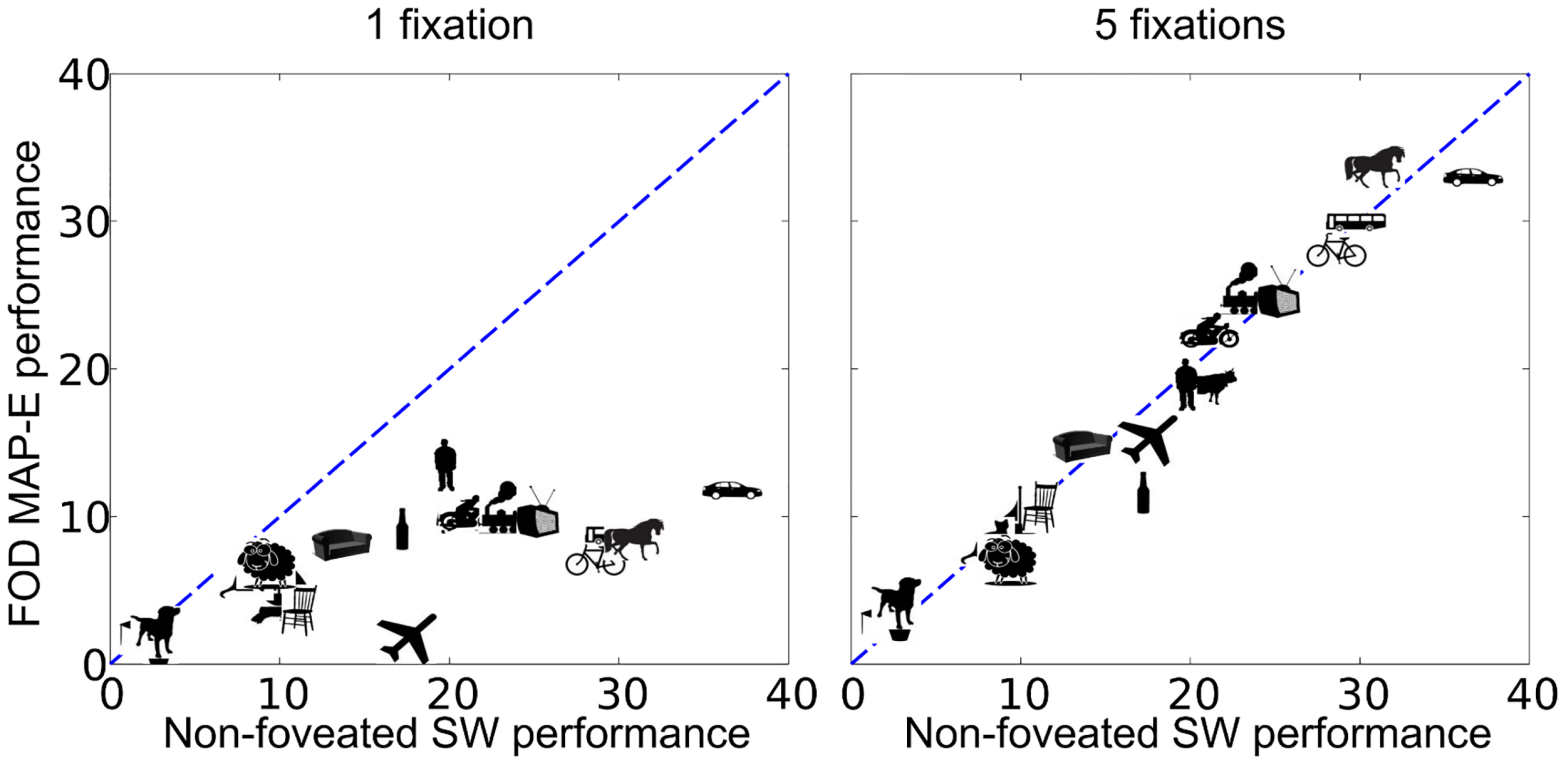
Area under the recall precision curve (AP scores) achieved by the non-foveated (SW) model and the foveated object detector with a Maximum a posteriori eye movement strategy and a starting fixation point to the side of the image (MAP-E). Symbols represent each object class type. Identity (diagonal) line corresponds to equal performance across models.

MAP-C performs well (84.8% relative performance) even with 1 fixation. The reason behind this result is the fact that, on average, bounding boxes in the PASCAL dataset cover a large portion of the images (average bounding box area normalized by image area is 0.2) and are located at and around the center [46]. To reduce the effects of these biases about the location of object placement on the results, we assessed the models with an initial fixation close to the edge of the image (MAP-E). When the initial fixation is closer to the edge of the image, performance is initially worse than when the initial fixation is at the center of the image. The difference in performance diminishes achieving similar performance with five fixations (0.2 difference in mean AP). Fig 5 shows how the distribution of AP scores for different object classes for MAP-E improves from 1 fixation to 5 fixations.

#### Importance of the guidance algorithm

To assess the importance of guided saccades towards the target, we compared performance of the MAP strategy against FOD that guides eye movements based on a random eye movement generator. Fig 4 allows comparisons of the relative performance of the MAP FOD and those with a random eye movement strategy. The performance gap between MAP-C, RAND-C pair and MAP-E, RAND-E pair highlights the performance costs of a foveated system without an algorithm to guide eye movements.

#### Computational cost savings

In both non-foveated SW based methods and the FOD, linear template evaluations, i.e. taking dot-products, is the main computationally costly operation. We define the computational cost of a method based on the total number of template evaluations (dot products) it executes (as also done in [47]). A model may have several templates with different sizes, so instead of counting each template evaluation as 1 operation, we take into account the dimensionalities of the templates. For example, the cost of evaluating a (6-cell)x(8-cell) HoG template is counted as 48 operations.

In order to compute the computational cost of a model, we run it on a subset of the test image set and count the total number of operations (as described above) actually performed. Note that, in order to compute a detection score, the FOD first performs a feature pooling (based on the location of the component in the visual field) and then a linear template evaluation. Since these are both linear operations, we combine them into the evaluation of a single template. This means that the costs of feature pooling and template evaluation are included in the evaluation of this single template.

The last column of Table 1 gives the computational costs of the non-foveated SW method and the FOD. For the FOD the computational cost is reported as a function of different number of fixations. For ease of comparison, we normalized the costs so that the non-foveated SW method performs 100 operations in total. The results show that FOD is computationally more efficient. FOD achieves almost the same accuracy performance—98.8% of the non-foveated SW’s average-precision score—at 49.6% of the computational cost of the non-foveated SW model. Typically, in computer vision, complexity of algorithms are specified in terms of the input image size. The computational complexity of the non-foveated model, in this sense, can be expressed easily. However, this is not the case for the FOD whose computational complexity does not depend on image size but on a number of factors including the scaling factor of pooling regions and the number of required fixations. For this reason, we compare the computational costs of the FOD and the non-foveated SW models in terms of the total number of actual dot-product operations performed in template evaluations.

#### Using richer object detection models at the fovea to increase performance

The FOD uses linear classifiers to detect objects. Here we evaluate the effects of using richer and more expensive classifiers but restricted only to the fovea. After each fixation, the FOD evaluated a full Deformable Parts Model (DPM) detector [28] only at foveal locations that score above a threshold which is determined on the training set to achieve high recall rate (95%). The DPM is a computer vision object detector that models not only the overall appearance of the object (via what they call the root filter) but also its parts. We refer to the new foveated object detector that uses DPM at its fovea as the “FOD-DPM”.

**Fig 6:**
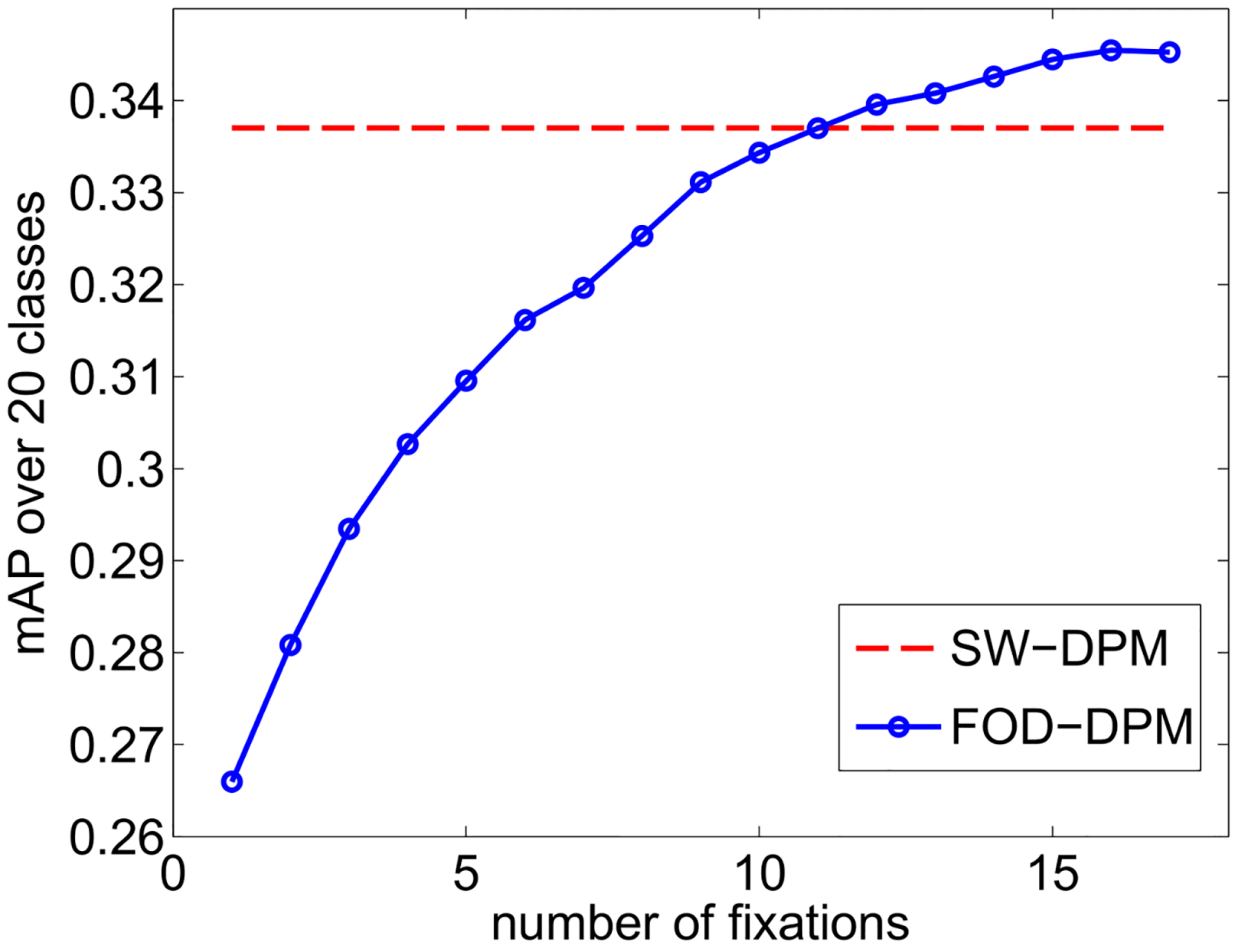
FOD-DPM’s performance (mean AP over all 20 classes) as a function of number of fixations. FOD-DPM achieves SW-DPM’s performance at 11 fixations and exceeds it with more fixations.

**Fig 7:**
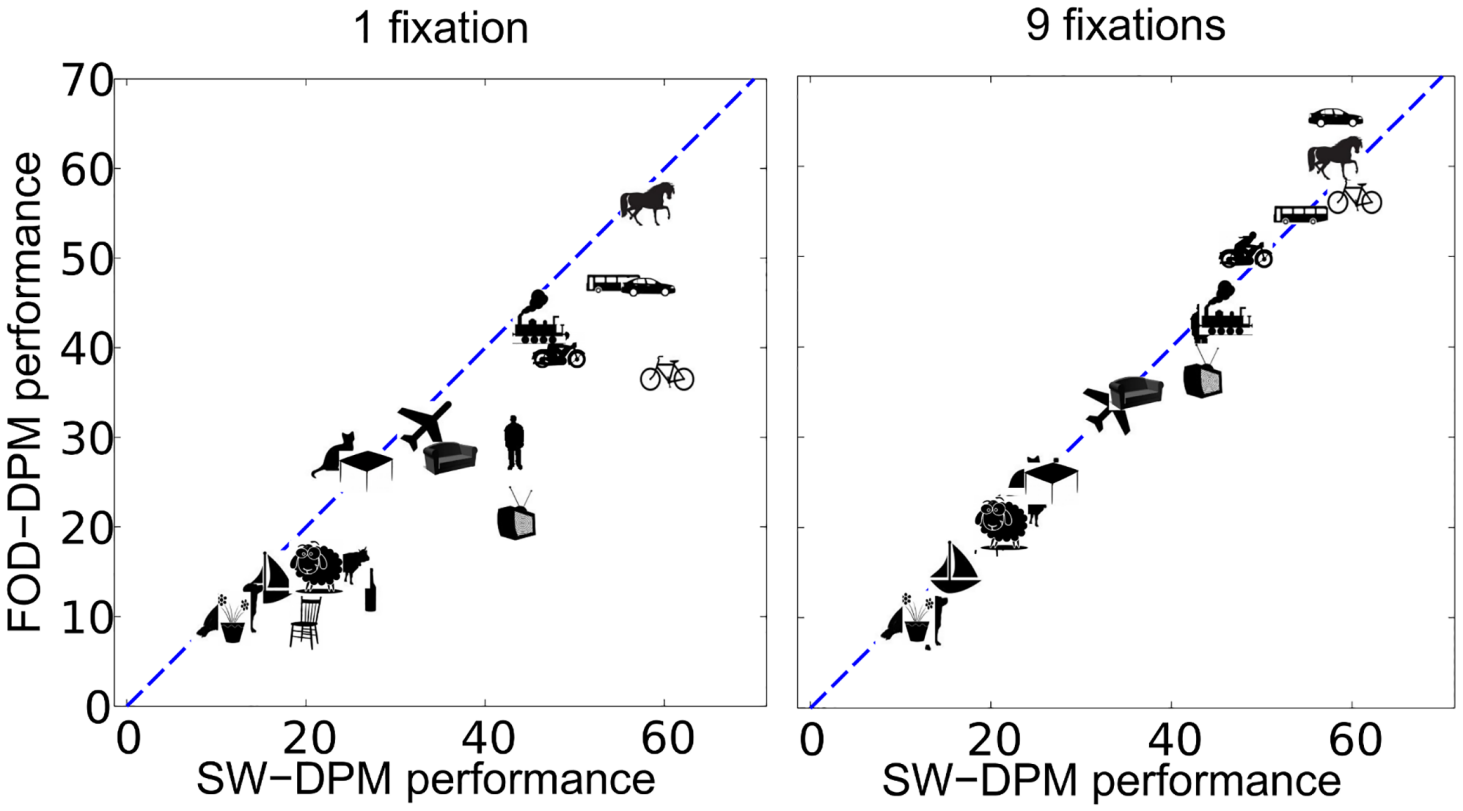
Per class AP scores achieved by FOD-DPM and non-foveated SW-DPM.

Table 2 and Fig 6 present the performance results of this approach and compares it to the non-foveated (sliding window) DPM model which we call the SW-DPM, for short. FOD-DPM achieves a similar average performance to that of SW-DPM (98.2% relative performance, 0.6 AP gap) using 9 fixations and exceeds DPM’s performance starting from 11 fixations. On some classes (e.g. bus, car, horse), FOD-DPM exceeds SW-DPM’s performance probably due to lesser number of evaluations and reduced false positives; on other cases (e.g. bike, dog, tv) FOD-DPM underperforms probably due to low recall rate of the FOD detector for these classes. Fig 7 shows AP scores of FOD-DPM and SW-DPM for each object class to demonstrate the improvement from 1 to 9 fixations.

**Table 2:** Per class percent average precision (AP), mean average precision (mAP) over all 20 classes and relative computational costs of FOD-DPM and DPM on the PASCAL VOC 2007 dataset. (Object class abbreviations are as follows. ap: aeroplane, bk: bike, bd: bird, bt:boat, bl: bottle, bs: bus, cr: car, ct: cat, ch: chair, cw: cow, dt: dining-table, dg: dog, hs: horse, mb: motorbike, pr: person, pt: potted-plant, sh: sheep, sf: sofa, tr: train, tv: tv-monitor).

		ap	bk	bd	bt	bl	bs	cr	ct	ch	cw	dt	dg	hs	mb	pr	pt	sh	sf	tr	tv	mAP	Cost
**SW-DPM**		33.2	60.3	10.2	16.1	27.3	54.3	58.2	23.0	20.0	24.1	26.7	12.7	58.1	48.2	43.2	12.0	21.1	36.1	46.0	43.5	33.7	100
**FOD-DPM**	**1**	31.0	37.1	10.0	14.3	12.9	47.1	46.7	28.0	9.3	15.5	26.2	10.7	56.0	39.7	29.4	9.8	15.5	27.6	43.4	21.5	26.6	0.46
	**5**	32.3	50.0	9.8	15.2	21.8	50.0	63.0	25.9	17.1	20.5	25.4	9.7	61.4	44.6	38.0	9.2	19.7	30.1	43.1	32.1	31.0	1.84
	**9**	33.2	56.6	9.9	15.6	25.3	54.6	65.3	25.3	19.8	22.0	24.9	9.4	60.9	50.8	41.7	10.0	20.4	34.9	44.3	37.3	33.1	3.09
	**13**	**33.4**	59.9	10.0	15.7	27.2	**54.8**	**65.7**	**25.0**	**20.5**	22.0	24.8	9.2	**62.0**	**51.9**	**44.5**	10.2	20.9	**36.8**	**46.2**	40.9	**34.1**	4.16

#### Computational savings of FOD-DPM

We compare the computational complexities of FOD-DPM and SW-DPM by their total number of operations as defined above. For a given object class, DPM model has 3 root filters and 8 6x6 part filters. It is straightforward to calculate the number of operations performed by SW-DPM as it uses the sliding window method. For FOD-DPM, the total number of operations is calculated by adding: 1) FOD’s operations and 2) SW-DPM’s operations at each high-scoring foveal detection bounding box ***b***, one DPM root filter (with the most similar shape as ***b***) and 8 parts evaluated at all locations within the boundaries of this root filter. Cost of feature extraction is not included as the two methods use the same feature extraction code. We report the computational costs of FOD-DPM and SW-DPM in the last column of Table 2. The costs are normalized so that SW-DPM’s cost is 100 operations. Results show that FOD-DPM drastically reduces the cost from 100 to 3.09 for 9 fixations. Assuming both methods are implemented equally efficiently, this would translate to an approximately **32x** speed-up. These results demonstrate the effectiveness of our foveated object detector in guiding the visual search. In the FOD-DPM implementation, the visual periphery has, in addition to the greater spatial pooling, much simpler processing relative to the fovea. The fovea has a subsequent parts processing that the periphery lacks. This is essential to account for much of the additional cost savings of the FOD-DPM vs. the simpler FOD model (compare the last columns of Tables 1 and 2). A qualitative difference in computations at the fovea and periphery is consistent with recent findings utilizing brief dichoptic presentation of visual stimuli and proposing more top-down processing at the fovea [48].

**Fig 8:**
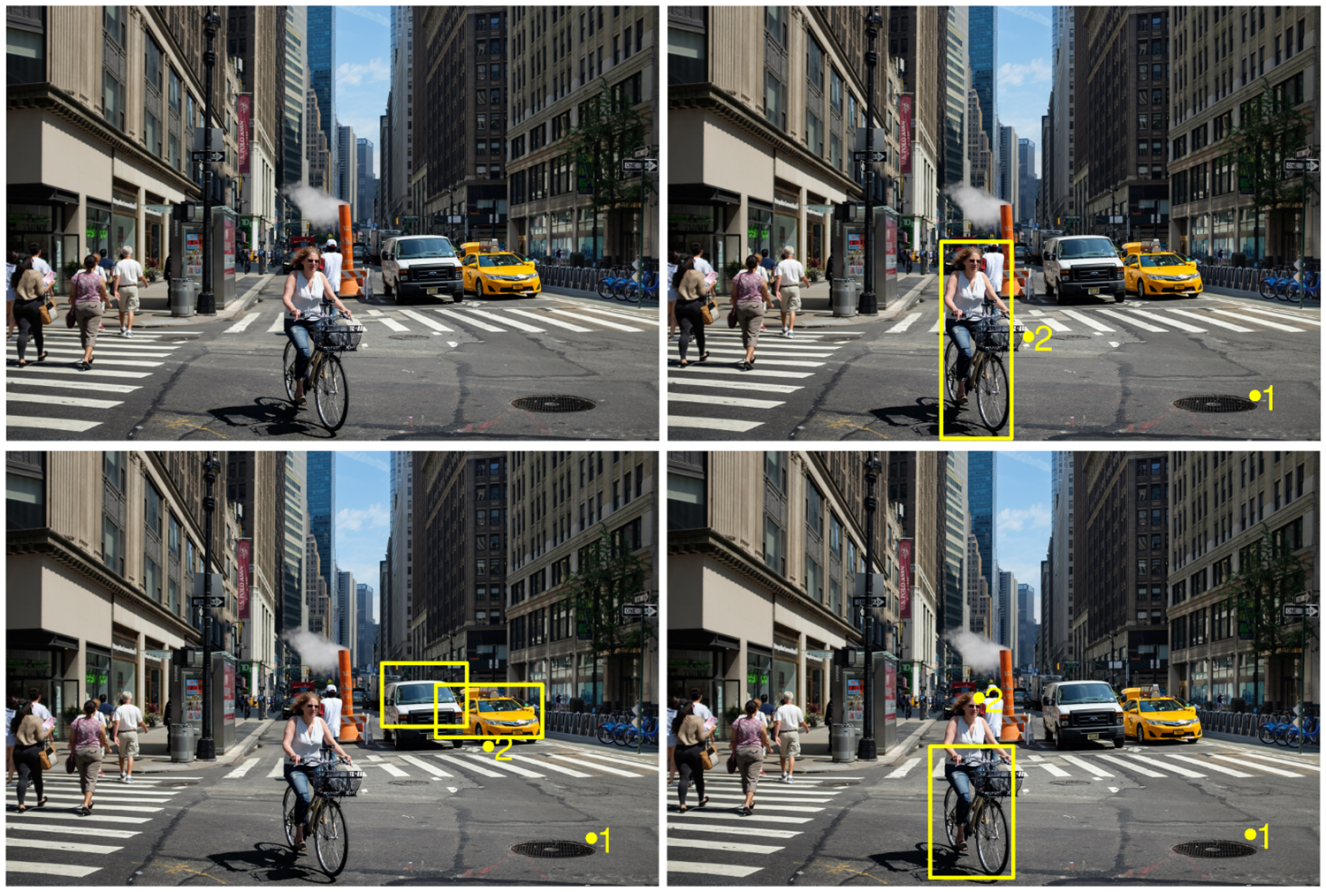
Fixation locations and bounding box predictions of the FOD for three different object classes (person, car and bicycle) but for the same image and initial point of fixation. Top-left: original image (source: https://www.flickr.com/photos/kristoffer-trolle/27882648666/ with Creative Commons license.), top-right: person detection, bottom-left: car detections, bottom-right: bicycle detection. Yellow dots show fixation points, numbers in yellow fonts indicate the sequence of fixations and the bounding boxes are the final detections.

Finally, Fig 8 shows sample detections by the FOD. We illustrate the trained bicycle, person and car models on an image outside of the PASCAL dataset. The models were assigned the same initial fixation location and we ran them for 3 fixations. Results show that the each model fixates at different locations, and these locations are attracted towards instances of the target objects being searched.

### Evaluation of the effect of foveation on saliency

Our previous sections suggest that a computationally less costly foveated system can achieve similar performance accuracy finding an object in real scenes as a system with homogeneous high spatial resolution. Research has shown that visual areas in the brain also rapidly compute bottom-up information in terms of salient regions defined by contrast, edges and color [5–7]. These salient regions serve to identify potential locations in scenes for further computation. The impact of a foveated visual system in such saliency computations is not known. Here, we evaluate whether identifying the most salient region in an image, an important component of bottom-up attention useful to identify potential regions of a scene for further scrutiny, is affected by the process of foveation. Or in the contrary, can a foveated system with eye movements identify the same salient regions with less computation than a non-foveated system?

We implemented a simple model of saliency that followed conceptually the model proposed by Li [6, 49, 50]. Such saliency model involves two computational aspects of saliency, namely iso-orientation suppression and contour enhancement, and also their dynamics. Here we only implemented the iso-orientation suppression aspect by using a simple center-surround operation.

The current simplified implementation of the saliency model first extracts features by convolving Gabor receptive fields (4 scales and 8 orientations) with the input image. Each cell pools (sums) Gabor responses per orientation, within its receptive field. Then, a center surround computation is implemented by subtracting each cell’s response by the pooled response of the neighboring cells at that same orientation (iso-orientation suppression). The spatial distribution of responses after the suppressions were considered the saliency map and the highest value the top saliency score. We implemented two versions of this saliency model, a non-foveated version consisting of only foveal cells and a foveated version that uses the simplified Freeman-Simoncelli model as its visual field (The same visual field that is used by the FOD.) (§Foveated visual field). The non-foveated saliency model processes all locations of the input image with the same (high) resolution and each Gabor receptive field was suppressed by pooling the Gabors with the same orientation at eight neighboring locations. The foveated model processes the input image with varying resolution. The foveal center surround suppression consisted of subtracting from a cell’s receptive field response the pooled activity across eight (nearest) surrounding cells of the same orientation. However, the peripheral center surround was implemented by subtracting the pooled responses across four nearest neighboring cells. The foveated saliency model makes eye movements based on the saliency values computed at the peripheral cells. It executes a saccade to the location with the maximum saliency value. After saccade execution, the saliency values around foveation (a 2-degree radius area around each fixation point) is inhibited (inhibition of return). This prevents the model from getting caught at a maximally salient location and not executing additional eye movements. After *n* fixations, the model integrates saliency values for each location across fixations and selects the top saliency location.

**Fig 9:**
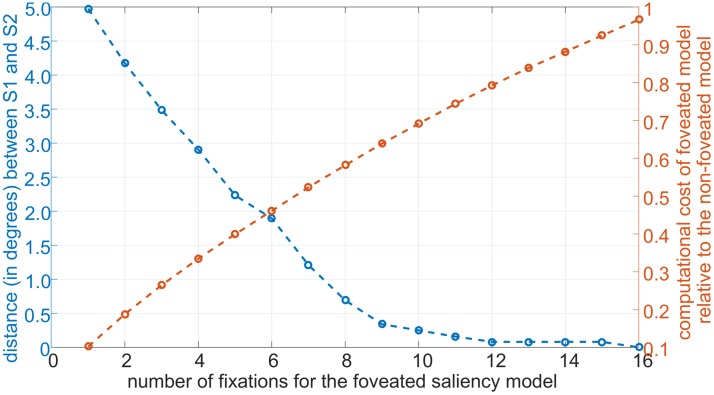
Performance comparison of the foveated saliency model versus the non-foveated saliency model. We ran both models for the simple task of identifying the topmost salient location, on 100 natural images randomly selected from the PASCAL VOC 2007 dataset. The blue curve plots the average distance (in degrees) between the topmost salient locations, S1 and S2, found by the foveated and the non-foveated model, respectively, on the same image. Note that this location is unique and fixed for the non-foveated model while it changes for the foveated model as the model explores the image, i.e. makes more and more fixations. The red curve plots the average number of iso-orientation suppression operations of the foveated model relative to that of the non-foveated model. Again, the number of such operations for the non-foveated model is fixed but it changes for the foveated model with the number of fixations. Foveated model finds the same topmost salient location as the non-foveated model, after 16 fixations. Notably, after 8 fixations, the distance between S1 and S2 becomes less than 1 degree. The foveated model achieves this level of accuracy by doing 42% less iso-orientation suppression operations than the non-foveated model.

We compared decisions of the non-foveated and foveated saliency models in terms of their agreement in selecting the top salient location within the image. We compared the computational costs of the two models in terms of their associated total number of center-surround operations. Fig 9 shows the distance in degrees between the top salient location from the non-foveated saliency model (blue line) and that of the foveated saliency model. The comparison is plotted as a function of increasing number of fixations for the foveated saliency model. In red we show the fraction of the number of center surround operations of the foveated saliency model relative to the non-foveated model. This is shown as a function of number of fixations. The results show that the foveated model can generate a similar prediction for the most salient region as the non-foveated model but with a significantly less number of center-surround operations. Mathematical details of the foveated saliency model can be found in the supplementary section Part E in S1 Text.

## Discussion

### The benefits of a foveated visual system

The objective of our current work was to evaluate within a common framework the accuracy costs and computational savings of a foveated visual system relative to a full high resolution system. We evaluated an object detection paradigm over 20 object classes from a standard object detection dataset. Our results show that with five exploratory fixations, the foveated method achieves nearly the same performance as the non-foveated (high resolution SW) method (Fig 5). The foveated achieved such accuracy with 49.6% of the sliding window method’s computational cost. Using a richer model (such as the Deformable Parts Model, DPM [28]) that selects potential locations for further object part processing, the foveated version of the model was able to match and even outperform the non-foveated SW-DPM while achieving computational savings (at less then **4.16%** of the SW-DPM’s computational cost). In addition, Fig 4 highlights the importance of guided eye movements in re-orienting the fovea to regions of interest in the scene. Eliminating the guidance diminishes the model’s ability to correctly detect the object with additional fixations. Together the results suggest that a foveated visual system with guided eye movements provide computational savings while preserving an organism’s ability to successfully detect objects in scenes. Our conclusions are limited by the utilized data set which although large does not represent all set of tasks that an organism or a human might face. For example, the PASCAL dataset does not contain a large number of images with small objects in the scenes. Such scenes might represent a more challenging test set for the foveated object detector and show potential accuracy losses beyond those quantified in the current investigation. On the other hand, our model only guides its eye movements based on peripheral information about the target while humans are known to utilize information about global statistics [12, 51] and object co-occurrence [14–16, 52] and multiple object configuration [53] to guide eye movements and aids perceptual decisions [54, 55]. Inclusion of such sources of scene information might improve the FOD’s accuracy.

In addition to evaluating performance of a foveated visual system for object search, we assessed the impact of a foveated visual system on the computation of saliency which is a fundamental bottom-up component that guides attention [5–7]. We compared a simplified implementation of a non-foveated saliency model and its foveated counterpart. The results showed that the foveated visual system with about 8-10 eye movements could approximate the same selection of the top salient location by a non-foveated high resolution saliency model. Future work should evaluate the generality of the results to more complex and different models of saliency [5–7, 56, 57]. In addition, our implementation of a foveated visual system concentrated on the spatial pooling losses occurring at cortical areas and possibly responsible for a great portion of the bottleneck of visual processing at the periphery [38]. A more detailed model could implement the peripheral loss starting with the reduction in photoreceptor density [35] and spatial sampling of retinal ganglion cells [58]. We believe that such detailed implementation would not qualitatively change the fundamental result of our paper, but future efforts need to test such prediction.

Our work assessed the impact of foveation on perceptual performance but does not address the mechanism by which the foveated visual system is evolved or shaped during development. An interesting theory contends that cone density is shaped by the probability distribution of objects across the retina and is thus influenced by the frequency and accuracy of eye movements [59].

### Comparison to other biologically inspired methods

There have been previous efforts, (e.g. [60]), on biologically inspired object recognition. However, most of such models do not have a foveated visual field and thus do not execute eye movements. More recent work has implemented biologically inspired search methods. In [19], a fixed, pre-attentive, low-resolution wide-field camera is combined with a shiftable, attentive, high-resolution narrow-field camera, where the pre-attentive camera generates saccadic targets for the attentive, high-resolution camera. The difference between this and our method is that while their pre-attentive system has the same coarse resolution everywhere in the visual field, our method, which is a model of the V1 layer of the visual cortex, has a varying resolution that depends on the radial distance to the center of the fovea. There have been previous efforts to create foveated search models with eye movements [17, 24, 25, 61]. Such models have been applied mostly to detect simple signals in computer generated noise [17, 24, 62] and used as benchmarks to compare against human eye movements and performance.

Other biologically inspired methods include the target acquisition model (TAM) [26, 63], the Infomax model [27] and artificial neural network based models [64, 65]. TAM is a foveated model and it uses the Scale Invariant Feature Transform (SIFT) features [66] for representation and utilizes a training set of images to learn the appearance of the target object. However, their evaluation did not include variability in object appearance due to scale and viewpoint, i.e. the object instances always appeared at the same size and viewpoints both in training and testing sets. Simply using SIFT features does not guarantee successful detection of objects appearing at different sizes. Furthermore, their evaluation involves placing the objects on a uniform background rather than real scenes such as in the current work. The Infomax model, on the other hand, can use any previously trained object detector and works on natural images, although they report results for face detection only and not for generic object detection. Critically, the Infomax’s foveated architecture is not based on physiology and uses non-biological rectangular pooling regions. We emphasize that any evaluation of the performance cost of a foveated visual system will depend critically on the parameters of the pooling regions with retinal eccentricity. Thus, an accurate assessment of the performance of a human/primate foveated visual system requires implementing a model which pooling regions are bio-inspired and based on physiological measurements.

Larochelle and Hinton [64], and Bazzani et al. [65] developed artificial neural network based models that have some sort of foveation. However, their application areas were different. Larochelle and Hinton [64] applied their model to image categorization and Bazzani et al. [65] applied their model to object tracking in videos.

Most importantly, none of these models have evaluated a biologically plausible foveated architecture relative to a high resolution scheme within a common theoretical framework to assess the potential performance loss of a system with a human foveated visual system and guided eye movements.

### Relation to current state of the art approaches in object detection

There has been substantial progress (e.g. [28–30, 47, 67–73]) in object detection research in recent years. However, humans, for now, are still unsurpassed in their ability to search for objects in visual scenes. The human brain relies on a variety of strategies. Object detection approaches have increasingly included some of the human strategies [19, 28, 60, 65, 74].

One remaining crucial difference between the human visual system and a modern object detector is that while humans process the visual field with decreasing resolution away [31–34] from the fixation point and make saccades to collect information, typical object detectors [28] scan all locations at the same resolution and repeats this at multiple scales.

The sliding window (SW) method is the dominant model of search in object detection. Efficient alternatives to sliding windows can be categorized in two groups: 1. methods aimed at reducing the number of locations (*m*), 2. methods aimed at reducing the number of object categories (*n*). Since typically *m* >> *n*, there are a larger number efforts in trying to reduce *m*, however, reducing the contribution of the number of object classes has recently been receiving increasing interest as search for hundreds of thousands of object classes has started to be tackled [69]. According to this categorization, our proposed FOD method falls into the first group as it is designed to locate object instances by making a set of sequential fixations where in each fixation only a sparse set of locations are evaluated. Thus our proposed FOD scheme might provide an alternative bio-inspired method to other proposed methods to reduce the number of evaluated locations. There are number of previously proposed methods to reduce the number of locations to be evaluated. One line of research is the branch-and-bound methods [75, 76] where an upper bound on the quality function of the detection model is used in a global branch and bound optimization scheme. Although the authors provide efficiently computable upper bounds for popular quality functions (e.g. linear template, bag-of-words, spatial pyramid), it might not be trivial to derive suitable upper bounds for a custom quality function. Our method, on the other hand, uses binary classification detection model and is agnostic to the quality function used.

Another line of research is the cascaded detection framework [77–79] where a series of cheap to expensive tests are done to locate the object. Cascaded detection is similar to our method in the sense that simple, coarse and cheap evaluations are used together with complex, fine and expensive evaluations. However, we differ with it in that it is essentially a sliding window method with a coarse-to-fine heuristic used to reduce the number of total evaluations. Another coarse-to-fine search scheme is presented in [80] where a set of low to high resolution templates are used. The method starts by evaluating the lowest resolution template—which is essentially a sliding window operation—and selecting the high responding locations for further processing with higher resolution templates. Our FOD method, too, uses a set of varying resolution templates; however, these templates are evaluated at every fixation instead of serializing their evaluations with respect to resolution.

In [47], a segmentation based method is proposed to yield a small set of locations that are likely to correspond to objects, which are subsequently used to guide the search in a selective manner. The locations are identified in an object class-independent way using an unsupervised multiscale segmentation approach. Thus, the method evaluates the same set of locations regardless of which object class is being searched for. In contrast, in our method, selection of locations to be foveated is guided by learned object class templates.

The method in [74], similar to ours, works like a fixational system: at a given time step, the location to be evaluated next is decided based on previous observations. However, there are important differences. In [74], only a single location is evaluated at a time step whereas we evaluate all template locations within the visual field at each fixation. Their method returns only one box as the result whereas our method is able to output many predictions.

Mathe et al. [81] proposed a search model that has foveation. However, this model does not have peripheral processing. The next fixation location is decided based on the history of foveal observations. A foveal observation corresponds to evaluating several regions in high (original) resolution (produced by a third-party segmentation algorithm) around the current fixation location.

Finally, in recent years, we have witnessed a surge of research in convolutional neural network (CNN) based object detection [70–73]. The new models have almost doubled the detection performance (34.1 mAP our result versus 59.9 mAP for Faster RCNN [70] on the same dataset). It is clear that the type of features we extract from images (i.e. HOG) limit FOD’s performance as indicated by the higher performance of neural network based models (e.g. Faster RCNN). The FOD’s performance would improve if CNN-features were used instead of HoGs (but note that it would not be neurobiologically consistent to pool CNN features which have been shown to compute features beyond V1 [82], using a V1 model [39]. Nevertheless, it is important that future work evaluate the cost of a foveated system within the context of CNN framework to assess whether the findings in the current paper generalize to that approach.

Also, the computational cost of the new CNN models is much higher compared to the DPM-like (e.g. HOG+SVM) motivating even further the development of alternatives to sliding window method. Thus, a number of region proposal methods including Selective Search [47], edge boxes [83], region proposal networks [70] have been proposed (see Hosang et al.’s work [84] for a review). While it is true that region proposal methods greatly reduce the number of evaluation candidates, whether they are better than sliding window classifiers (in terms of accuracy and computational savings) is not a settled debate. State-of-the-art object detection (RCNN [85], Fast RCNN [86], Faster RCNN [70]) has abandoned region proposal methods. Faster RCNN, the best available object detector known to us, is not using a region proposal method to generate evaluation candidates. Instead, it uses a sliding window classifier which they call the “Region Proposal Network (RPN).” RPN slides a 3x3 window on the output of the topmost convolutional layer, evaluates 9 different hypothesis (3 scales, 3 aspect ratios) at each location, and outputs the best scoring 300 hypothesis as the object candidates. Theoretically, RPN itself could potentially be made faster by using a foveated method such as ours.

### Conclusion

To summarize, the findings show that a foveated architecture with guided eye movements can preserve both bottom-up saliency and top-down search for objects of a homogeneous high resolution system while incurring important computational cost savings. The findings might suggest a possible explanation for the evolution of a foveated visual system with eye movements as a possible solution that gives the organism similar ability to that of a non-foveated high resolution system but with decreased metabolic costs for the brain as well as reduced neural resource allocation.

## Methods and models

### Foveated visual field

The Freeman-Simoncelli (FS) model [39] is a neuronal population model of V1 and V2 layers of the visual cortex. The model specifies how responses are pooled (averaged together) hierarchically beginning from the lateral geniculate nucleus to V1 and then the V2 layer. V1 cells encode information about local orientation and spatial frequency whereas the cells in V2 pools V1 responses non-linearly to achieve selectivity for compound features such as corners and junctions. The model is based on findings and physiological measurements of the primate visual cortex and specifies the shapes and sizes of the receptive fields of the cells in V1 and V2. According to the model, the sizes of receptive fields increase linearly as a function of the distance from the fovea and this rate of increase in V2 is larger than that of V1, which means V2 pools larger areas of the visual field in the periphery. The reader is referred to [39] for further details.

We simplify the FS model in two ways. First, the model uses a Gabor filter bank to compute image features and we replace these with the HOG features [28, 41]. Second, we only use the V1 layer and leave the non-linear pooling at V2 as future work. We use this simplified FS model as the foveated visual field of our object detector which is shown in Fig 1. The fovea subtends a radius of 2 degrees. We also only simulate a visual field with a radius of 10 degrees which is sufficient to cover the test images presented at a typical viewing distance of 40 cm. The square boxes with white borders (Fig 1 represent the pooling regions within the fovea. The surrounding colored regions are the peripheral pooling regions. While the foveal regions have equal sizes, the peripheral regions grow in size as a function—which is specified by the FS model—of their distance to the center of the fovea. The color represents the weights that are used in pooling, i.e. weighted summation of, the underlying responses. A pooling region partly overlaps with its neighboring pooling regions (see the supplementary material of [39] for details). Specifically, (i) spatial weights of the pooling regions, (ii) locations of pooling regions, and (iii) the number of angle and eccentricity bins, and (iv) the scaling factor of the pooling regions with eccentricity in our FOD model are all directly based on the FS model’s V1 layer. Assuming a viewing distance of 40cm, the whole visual field covers about a 500x500 pixel area (a pixel subtends 0.08°). The foveal radius is 52 pixels subtending a visual angle of 4 degrees.

#### Feature pooling

First, HoG features are extract from the input image (see Fig 2). Then, we center the visual field around the current fixation point. At the fovea, where the pooling regions are 8x8 pixels, we directly use the HoG features, and in the periphery, each pooling region takes a weighted sum of HoG features of the 8x8 regions that are covered by that pooling region.

### The foveated object detector (FOD)

The model *M* consists of the application of *n* retino-specific, linear templates (i.e. classifiers) corresponding to different object viewpoints and resolutions. Thus, the model has *n* components, each of which consists of a linear template and its specific location vector:

$$\begin{array}{c} \text{Model}\;\;M^{\prime }s\;\;\text{parameters}:\;\;\left\{\left(w_{i},\ell_{i}\right):i=1,2,\ldots ,n\right\} \end{array}$$ (1)

where **w***_i_* is a linear template and ***ℓ***_*i*_ is the location of the template with respect to the center of the visual field. Among these parameters, **w***_i_* are learnable (given a dataset) but ***ℓ***_*i*_ are fixed (more on this in §“Initialization”) given the visual field parameters. The output of *M* given a fixation point *f* is an array of detection scores produced by the *n* retino-specific classifiers, corresponding to different locations within the image.

**Fig 10:**
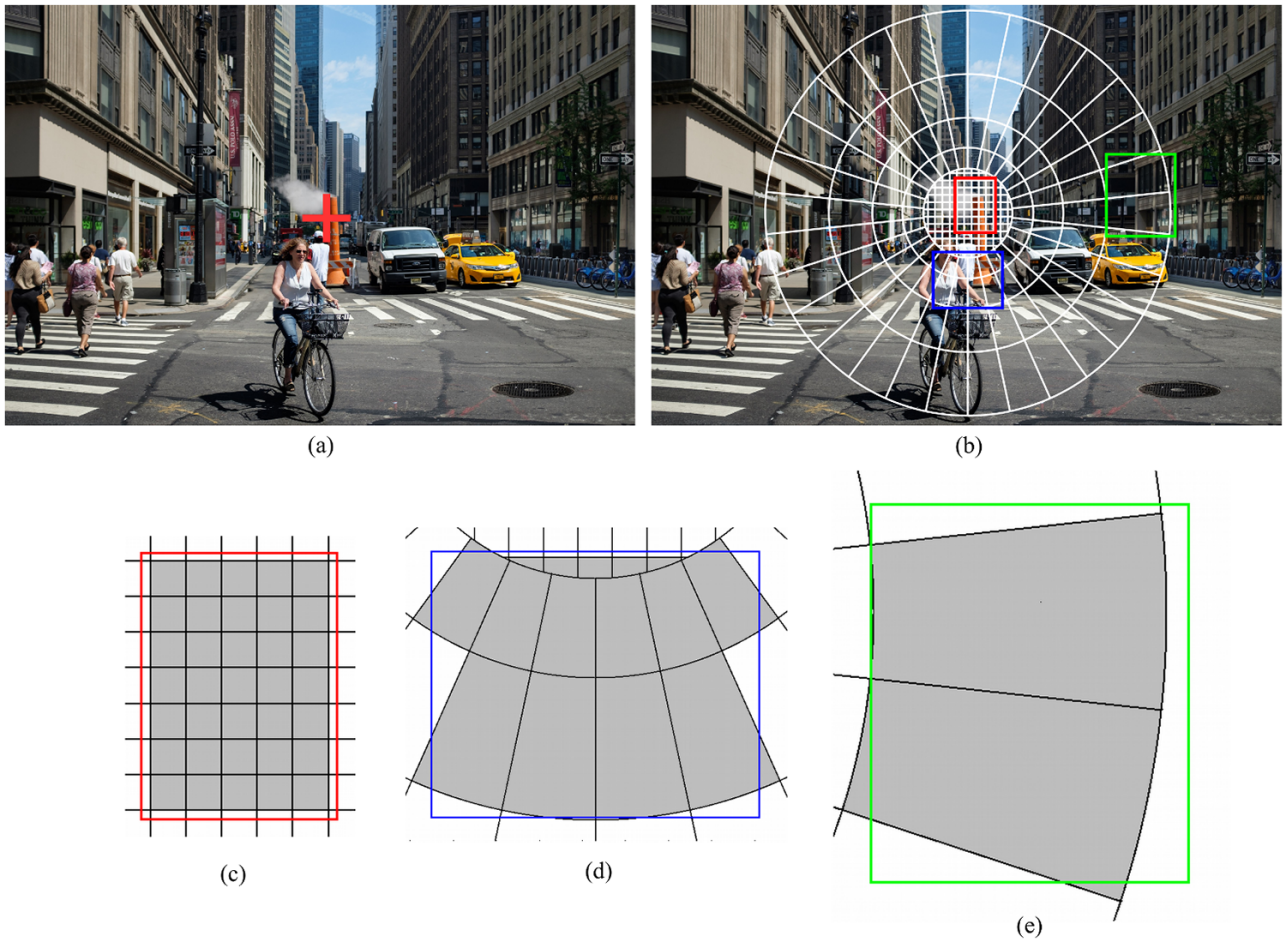
Illustration of the visual field of the model. (a) The model is fixating at the red cross mark on the image (see Fig 8’s caption for the source of the image). (b) Visual field (Fig 1) overlaid on the image, centered at the fixation location. White line delineate the borders of pooling regions. Nearby pooling regions do overlap. The weights (Fig 1) of a pooling region sharply decrease outside of its shown borders. White borders are actually iso-weight contours for neighboring regions. Colored bounding boxes show the templates of three components on the visual field: red, a template within the fovea; blue and green, two peripheral templates at 2.8 and 7 degree periphery, respectively. (c, d, e) Zoomed in versions of the red (foveal), blue (peripheral) and green (peripheral) templates. The weights of a template, **w***_i_*, are defined on the gray shaded pooling regions.

The location variable ***ℓ***_*i*_ defines a unique bounding box within the visual field for the *i*^*th*^ template. Specifically, ***ℓ***_*i*_ = (*ω*_*i*_, *h*_*i*_, *x*_*i*_, *y*_*i*_) is a vector whose variables respectively denote width, height and *x*, *y* coordinates of the *i*^*th*^ template within the visual field. The template, **w***_i_*, is a matrix of weights on the features extracted from the pooling regions underlying the bounding box ***ℓ***_*i*_. The dimensionality of **w***_i_*, i.e. the total number of weights, depends both on the width and height of its bounding box and its location in the visual field. A component within the fovea covers a larger number of pooling regions compared to a peripheral component with the same width and height, hence the dimensionality of a foveal template is larger. Three example components are illustrated in Fig 10 where the foveal component (red) covers 7x5 = 35 pooling regions while the (blue and green) peripheral components cover 15 and 2 regions, respectively. Since a fixed number of features is extracted from each pooling region (regardless of its size), foveal components have higher-resolution templates associated with them. We use the feature extraction implementation of DPM (rel5) [28, 87].

#### Detection model

Suppose that we are given a model *M* that is already trained for a certain object class. The model is presented with an image *I* and assigned an initial fixation location ***f***. We are interested in searching for an object instance in *I*. Because the size of a searched object is not known apriori, the model has to analyze the input image at various scales (the image is scaled up and down at several levels, and the whole feature extraction and template evaluation process are repeated per scale). It would be more desirable to utilize different size templates rather than scaling the input image. The reason we chose to scale the image to calculate the various templates is to equate this aspect of the FOD to the available high resolution SW model, i.e. the Deformable Parts Model (DPM) [28]. If we had chosen to train the FOD by using different template size, then the performance comparison against the existing SW model would have been a function of both foveation and any difference in object detection classifiers. This would have hindered the assessment of the isolated effect of foveation. While not perfect, it was the best solution to achieve the goals of the paper.

We use the same set of image scales given in [28] and use *σ* to denote a scale from that set. When used as a subscript to an image, e.g. *I*_*σ*_, it denotes the scaled version of that image, i.e. width (and height) of *I*_*σ*_ is *σ* times the width (and height) of *I*. *σ* also applies to fixation locations and bounding boxes: if ***f*** denotes a fixation location (*f*_*x*_, *f*_*y*_), then ***f***_*σ*_ = (*σf*_*x*_, *σf*_*y*_) (i.e. ***f***_*σ*_ is a vector containing two elements: *σ* times *f*_*x*_ and *σ* times *f*_*y*_); for a bounding box ***b*** = (*w*, *h*, *x*, *y*), ***b***_*σ*_ = (*σw*, *σh*, *σx*, *σy*).

To check whether an arbitrary bounding box ***b*** within *I* contains an object instance, while the model is fixating at location f, we compute a detection score as

$$\begin{array}{c} s\left(I,b,f\right)=\underset{\sigma }{\text{max}}\underset{c\in G(b_{\sigma },f_{\sigma })}{\text{max}}\;w^{T}\Psi \left(I_{\sigma },f_{\sigma },c\right) \end{array}$$ (2)

where Ψ(*I*_*σ*_, ***f***_*σ*_, *c*) is a feature extraction function which returns the features of *I*_*σ*_ for component *c* (see Eq (1)) when the model is fixating at ***f***_*σ*_. The vector **w** is the blockwise concatenation of the templates of all components. Ψ(⋅) effectively chooses which component to use, that is $w^{T}\Psi (I_{\sigma },f_{\sigma },c)=w_{c}^{T}\Psi (I_{\sigma },f_{\sigma },c)$. The fixation location, ***f***_*σ*_, together with the component *c* define a unique location, i.e. a bounding box, on *I*_*σ*_. *G*(***b***_*σ*_, ***f***_*σ*_) returns the set of all components whose templates have a predetermined overlap (intersection over union should be at least 0.7 as in [28]) with ***b***_*σ*_ when the model is fixating at ***f***_*σ*_. During both training and testing, *σ* and *c* are latent variables for example (*I*, ***b***).

Ideally, *s*(*I*, ***b***, ***f***) > 0 should hold for an appropriate ***f*** when *I* contains an object instance within ***b***. For an image that does not contain an object instance, *s*(*I*, ***b*** = ∅, ***f***)<0 should hold for any ***f***. For this to work, a subtlety in *G*(⋅)’s definition is needed: *G*(∅, ***f***) returns all components of the model (Eq (1)). During training, this will enforce the responses of all components for a negative image to be suppressed down.

#### Integrating observations across multiple fixations

So far, we have looked at the situation where the model has made only one fixation. We describe in Section Eye movement strategy how the model chooses the next fixation location. For now, suppose that the model has made *m* fixations, ***f***_1_, ***f***_2_, …, ***f***_*m*_, and we want to find out whether an arbitrary bounding box ***b*** contains an object instance. This computation involves integrating observations across multiple fixations, which is a considerably more complicated problem than the single fixation case. The Bayesian decision on whether ***b*** contains an object instance is based on the comparison of posterior probabilities:

$$\begin{array}{c} \frac{P(y_{b}=1|f_{1},f_{2},\ldots ,f_{m},I)}{P(y_{b}=0|f_{1},f_{2},\ldots ,f_{m},I)}{\;}_{>}^{<}1 \end{array}$$ (3)

where *y*_***b***_ = 1 denotes the event that there is an object instance at location ***b***. We use the posteriors’ ratio as a detection score, the higher it is the more likely ***b*** contains an instance. Computing the probabilities in (3) requires training a classifier per combination of fixation locations for each different value of ***m***, which is intractable. We approximate it using a conditional independence assumption (for the derivation, see Part C in S1 Text):

$$\begin{array}{c} \frac{P(y_{b}=1|f_{1},f_{2},\ldots ,f_{m},I)}{P(y_{b}=0|f_{1},f_{2},\ldots ,f_{m},I)}\approx \prod_{i=1}^{m}\frac{P(y_{b}=1|f_{i},I)}{P(y_{b}=0|f_{i},I)}. \end{array}$$ (4)

We model the probability *P*(*y*_***b***_ = 1|***f***, *I*) using a classifier and use the sigmoid transfer function to convert raw classification scores to probabilities:

$$\begin{array}{c} P\left(y_{b}=1|f,I\right)=\frac{1}{1+e^{\left(-s(I,b,f)\right)}}. \end{array}$$ (5)

We simplify the computation in (4) by taking the log (for the derivation, see Part D in S1 Text):

$$\begin{array}{c} \text{log}(\prod_{i=1}^{m}\frac{P(y_{b}=1|f_{i},I)}{P(y_{b}=0|f_{i},I)})=\sum_{i=1}^{m}s\left(I,b,f_{i}\right). \end{array}$$ (6)

Taking the logarithm of posterior ratios does not alter the ranking of detection scores for different locations, i.e. ***b***’s, because logarithm is a monotonic function. In short, the detection score computed by the FOD for a certain location ***b***, is the sum of the individual scores for ***b*** computed at each fixation.

After evaluating (6) for a set of candidate locations, final bounding box predictions are obtained by non-maxima suppression [28], i.e. given multiple predictions for a certain location, all predictions except the one with the maximal score are discarded.

### Eye movement strategy

We use the maximum-a-posteriori (MAP) model [43] with inhibition of return (see next subsection) as the basic eye movement strategy of the FOD. The MAP model selects the location with the highest posterior probability of containing the target object as the next fixation location, that is ***f***_*i*+1_ = center of ℓ* where

$$\begin{array}{c} \ell^{*}=\underset{\ell }{\arg\;\max}\;P\left(y_{\ell }=1|f_{1},f_{2},\ldots ,f_{i},I\right). \end{array}$$ (7)

This search is done over uninhibited locations only. Finding the maximum of the posterior above is equivalent to finding the maximum of the posterior ratios,

$$\begin{array}{c} \underset{\ell }{\arg\;\max}\;P\left(y_{\ell }=1|f_{1},\ldots ,f_{i},I\right)=\underset{\ell }{\arg\;\max}\;\frac{P(y_{\ell }=1|f_{1},\ldots ,f_{i},I)}{P(y_{\ell }=0|f_{1},\ldots ,f_{i},I)} \end{array}$$ (8)

since for two arbitrary locations ***ℓ***_1_, ***ℓ***_2_; let *p*_1_ = *P*(*y*_***ℓ***_1__ = 1|⋅) and *p*_2_ = *P*(*y*_***ℓ***_2__ = 1|⋅), then we have

$$\begin{array}{c} \frac{p_{1}}{1-p_{1}}>\frac{p_{2}}{1-p_{2}}\Rightarrow p_{1}>p_{2}. \end{array}$$ (9)

### Inhibition of return

After each fixation, a circular area with approximately 2 degree radius around the fixation location is inhibited. The model is not allowed to fixate to a previously inhibited location.

### Training the model

#### Initialization

**Fig 11:**
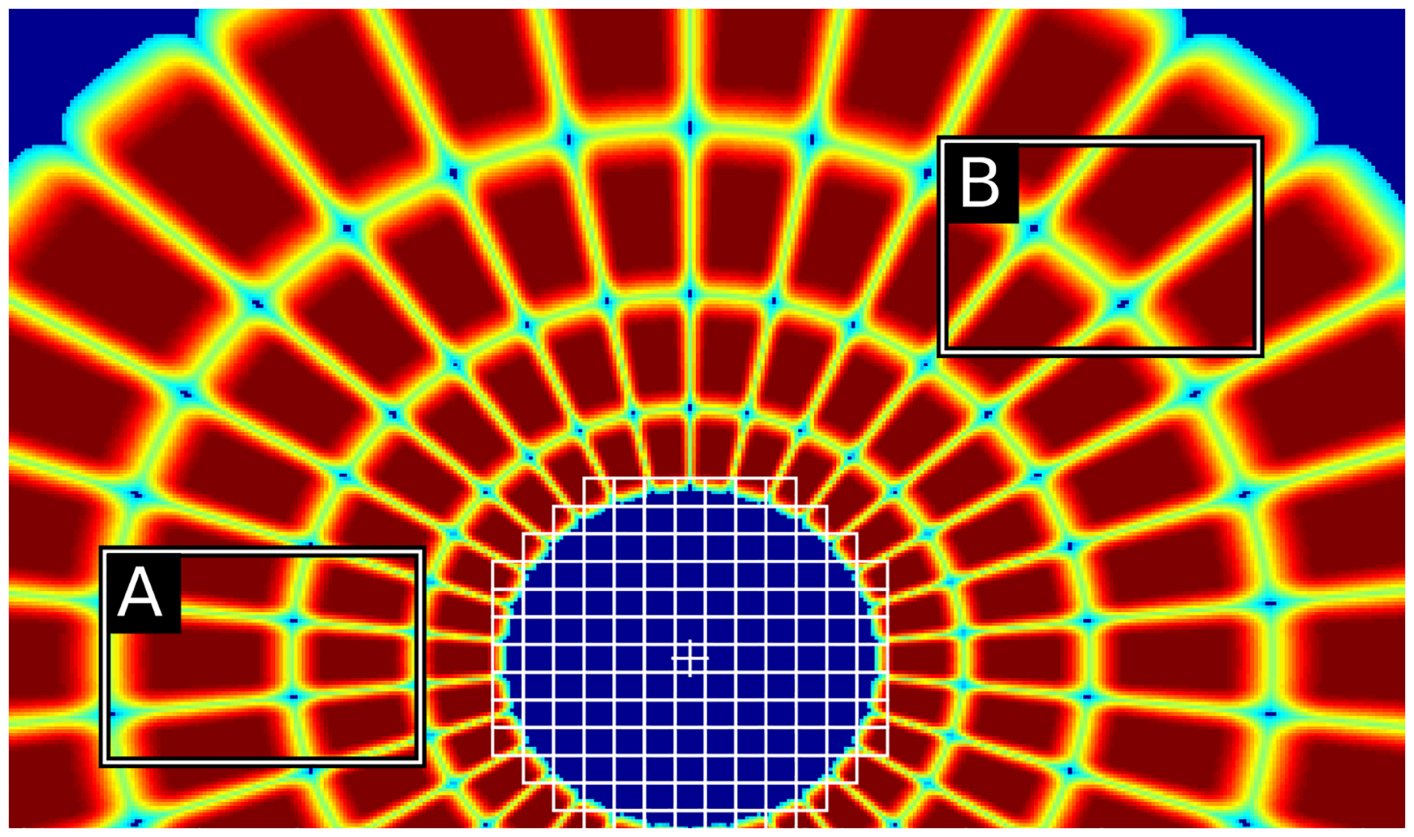
Two bounding boxes (A, B) are shown on the visual field. While box A covers a large portion of the pooling regions that it intersects with, box B’s coverage is not as good. Box B is discarded as it does not meet the overlap criteria (see text), therefore a component for B in the model is not created.

A set of dimensions (width and height) is determined from the bounding box statistics of the examples in the training set as done in the initialization of the DPM model [28]. Then, for each width and height, new components with these dimensions are created to tile the entire visual field. However, the density of components in the visual field is not uniform. Locations, i.e. bounding boxes, that do not overlap well with the underlying pooling regions are discarded. To define goodness of overlap, a bounding box is said to intersect with an underlying pooling region if more than one fifth of that region is covered by the bounding box. Overlap is the average coverage across the intersected regions. If the overlap is more than 75%, then a component for that location is created, otherwise the location is discarded (see Fig 11 for an example). In addition, no components are created for locations that are outside of the visual field. Weights of the component templates (**w***_i_*) are initialized to arbitrary values. Training the model is essentially optimizing these weights on a given dataset.

#### Training

Consider a training set $D={\left\{\left(I_{i},b_{i}\right)\right\}}_{i=1}^{K}$ where *I*_*i*_ is an image and ***b***_*i*_ a bounding box and *K* is the total number of examples. If *I*_*i*_ does not contain any positive examples, i.e. object instances, then ***b***_*i*_ = ∅. Following the DPM model [28], we train model templates using a latent-SVM formulation:

$$\begin{array}{c} \underset{w}{\arg\min}\;\frac{1}{2}{\left||w|\right|}_{2}^{2}+C\sum_{i=1}^{K}\sum_{f\in F(I_{i},b_{i})}\text{max}\left(0,1-y_{i}s\left(I_{i},b_{i},f\right)\right). \end{array}$$ (10)

where *y*_*i*_ = 1 if ***b***_*i*_ ≠ ∅ and *y*_*i*_ = −1, otherwise. The set ***f***(*I*_*i*_, ***b***_*i*_) denotes the set of all *feasible* fixation locations for example (*I*_*i*_, ***b***_*i*_). For ***b***_*i*_ ≠ ∅, a fixation location is considered feasible if there exists a model component whose bounding box overlaps with ***b***_*i*_. For ***b***_*i*_ = ∅, all possible fixation locations on *I*_*i*_ are considered feasible.

Optimizing the cost function in (10) is manageable for mixtures with few components, however, the FOD has a large number of components in its visual field (typically, for an object class in the PASCAL VOC 2007 dataset [40], there are around 500–700) and optimizing this cost function becomes prohibitive in terms of computational cost. As an alternative, cheaper linear classifiers can be used. Recently, linear discriminant analysis (LDA) has been used in object detection ([42]) producing surprisingly good results with much faster training time. Training a LDA classifier amounts to computing Σ^−1^(*μ*_1_−*μ*_0_) where *μ*_1_ is the mean of the feature vectors of the positive examples, *μ*_0_ is the same for the negative examples and Σ is the covariance matrix of these features. Here, the most expensive computation is the estimation of Σ, which is required for each template with different dimensions. However, it is possible to estimate a global Σ from which covariance matrices for templates of different dimensions can be obtained [42]. For the FOD, we estimate the covariance matrices for the foveal templates and estimate the covariance matrices for peripheral templates by applying the feature pooling transformations to the foveal covariance matrices.

We propose to use LDA in a latent-SVM-like framework as an alternative to the method in [42] where positive examples are clustered first and then a LDA classifier is trained per cluster. Consider the *t*^*th*^ template, **w***_t_*. LDA gives us that LDA gives us that $w_{t,\text{LDA}}=\Sigma_{t}^{-1}\left(\mu_{t}^{\text{pos}}-\mu_{t}^{\text{neg}}\right)$ where Σ_*t*_ is the covariance matrix for template *t*, $\mu_{t}^{\text{pos}}$ and $\mu_{t}^{\text{neg}}$ are the mean of positive and negative feature vectors, respectively, assigned to template *t*. We propose to apply an affine transformation to the LDA classifier:

$$w_{t}=\left[\begin{array}{lllll} \alpha_{t} & & & & \\ & \alpha_{t} & & 0 & \\ & & \ddots & & \\ & 0 & & \alpha_{t} & \\ & & & & \beta_{t} \end{array}\right]\left[\begin{array}{c} w_{t,\text{LDA}}\\ 1 \end{array}\right]=\left[\begin{array}{c} \alpha_{t}w_{t,\text{LDA}}\\ \beta_{t} \end{array}\right]$$ (11)

and modify the cost function as

$$\underset{\alpha ,\beta }{\text{arg}\;\text{min}}\left(\frac{1}{2}{\left|\left|w\right|\right|}_{2}^{2}+C\sum_{t=1}^{N}\text{max}\left(0,1+w_{t}^{T}\mu_{t}^{neg}\right)+C\sum {}_{{}_{{}_{i\in \{i|b_{i}\neq \emptyset \}}}}\sum {}_{{}_{f\in F\left(I_{i},b_{i}\right)}}\text{max}\left(0,1-y_{i}s\left(I_{i},b_{i},f\right)\right)\right)$$ (12)

where the first summation pushes the score of the mean of the negative examples to under zero and the second summation, taken over positive examples only, pushes the scores to above 0. ***α*** and ***β*** are appropriate blockwise concatenation of *α*_*t*_ and *β*_*t*_s. *C* is the regularization constant. Overall, this optimization effectively calibrates the dynamic ranges of different templates’ responses in the model so that the scores of positive examples and negative means are pushed away from each other while the norm of **w** is constraint to prevent overfitting. This formulation does not require the costly mining of hard-negative examples of latent-SVM. We call this formulation (Eq 12) as latent-LDA.

To optimize (12), we use the classical coordinate-descent procedure. We start by initializing **w** by training on warped-positive examples as in [28]. Then, we alternate between choosing the best values for the latent variables while keeping **w** fixed, and optimizing for **w** while keeping the latent variables of positive examples fixed.

## Supporting information

S1 Text Supporting information for various sections. S1 Text contains a sample recall-precision curve, comparison of sliding-window based methods, derivations for Eqs (4) and (6), details of the foveated saliency model and comments on the effects of inhibition-of-return on performance.
(PDF)
