# Supplementary material for: Object detection through search with a foveated visual system
Source: PLoS Comput Biol. 2017 Oct 9;13(10):e1005743. doi: 10.1371/journal.pcbi.1005743 (PMC5669499; doi:10.1371/journal.pcbi.1005743)
Supplement: S1 Text — contains a sample recall-precision curve, comparison of sliding-window based methods, derivations for Eqs (4) and (6), details of the foveated saliency model and comments on the effects of inhibition-of-return on performance. (PDF) [file pcbi.1005743.s001.pdf]

S1 Supporting Information

[A] An example recall-precision curve Fig 1 shows the performance of the FOD using the MAP eye movement rule with 5 fixations, on the "bus" object class. Area under the curve, i.e. average precision, is 0.31 or 31%.

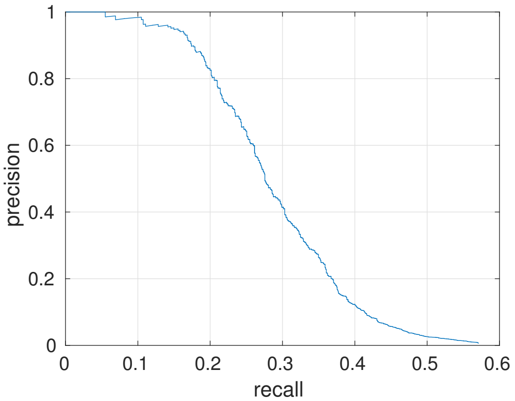

Fig 1. An example recall-precision plot.

[B] Comparison of SW based methods We compared our SW implementation, which corresponds to using foveal templates only, to three state-of-the-art methods that are also SW based [1–3]. Table 1 gives the AP (average precision) results, i.e. area under the precision-recall curve per class, and mean AP (mAP) over all classes. Originally, the deformable parts model (DPM) uses object parts, however, in order to make a fair comparison with our model, we disabled its parts. The first row of Table 1 shows the latest version of the DPM system [4] with the parts-learning code disabled. The second row shows results for another popular SVM-based system, known as the exemplar-SVM (E-SVM), which also only models whole objects, not its parts. Finally, the third row shows results from a LDA-based system, "discriminative decorrelation for classification" (DCC) [3]. All three systems are based on HOG features and mixture of linear templates. The results show that SVM based systems perform better than the LDA based systems, which is not a surprising finding since it is well known that discriminative models outperform generative models in classification tasks. However,

Table 1. Per class percent average precision (AP) and mean average precision (mAP) over all 20 classes of SW based methods on the PASCAL VOC 2007 dataset.

	aero	bike	bird	boat	bottle	bus	car	cat	chair	cow	table	dog	horse	mbike	person	plant	sheep	sofa	train	tv	mAP
**DPM [1]**	23.6	48.6	9.7	11.0	19.3	40.4	45.2	12.4	15.4	19.4	17.4	4.0	44.7	36.4	31.2	10.9	14.1	19.5	32.2	37.0	24.6
**E-SVM [2]**	20.4	40.7	9.3	10.0	10.3	31.0	40.1	9.6	10.4	14.7	2.3	9.7	38.4	32.0	19.2	9.6	16.7	11.0	29.1	31.5	19.8
**DCC [3]**	17.4	35.5	9.7	10.9	15.4	17.2	40.3	10.6	10.3	14.3	4.1	1.8	39.7	26.0	23.1	4.9	14.1	8.7	22.1	15.2	17.1
**Our SW**	17.5	28.6	9.7	10.4	17.3	29.8	36.7	7.9	11.2	21.0	2.3	2.7	30.9	21.1	19.7	3.0	9.2	13.7	23.5	25.2	17.1

LDA's advantage against this performance loss is that it is ultra fast to train, which is exactly the reason we chose to use LDA instead of SVM. Once the background covariance matrices are estimated (which can be done once and for all [3]), training is as easy as taking the average of the feature vectors of positive examples and doing a matrix multiplication. We estimated the time that training a SVM based system for our FOD to be about 300 hours (approximately 2 weeks) for a single object class, whereas the LDA based system can be trained under an hour on the same machine which has an Intel i7 processor.

Although our SW method achieves the same mean AP (mAP) score as the DCC method [3], the latter has a detection model with higher computational cost. We use 2 templates per class while DCC trains more than 15 templates per class within an exemplar-SVM [2]-like framework. DCC considers the dot product of the feature vector of the detection window with every exemplar within a cluster, which basically means that a detection window is compared to all positive examples in the training set. In our case, the number of dot products considered per detection window is equal to the number of templates, which is 2 in this paper, which clearly demonstrates the advantage of our latent-LDA approach over DCC [3].

[C] Approximation of the Bayesian decision

Derivation for Equation (4):

$$\frac{P(y_{\boldsymbol{b}}=1|\boldsymbol{f}_1,\ldots,\boldsymbol{f}_m,I)}{P(y_{\boldsymbol{b}}=0|\boldsymbol{f}_1,\ldots,\boldsymbol{f}_m,I)} = \frac{P(f_1,\ldots,\boldsymbol{f}_m|y_{\boldsymbol{b}}=1,I)P(y_{\boldsymbol{b}}=1|I)}{P(f_1,\ldots,\boldsymbol{f}_m|y_{\boldsymbol{b}}=0,I)P(y_{\boldsymbol{b}}=0|I)} \tag{1}$$

$$\approx \prod_{i=1}^{m}\frac{P(\boldsymbol{f}_i|y_{\boldsymbol{b}}=1,I)}{P(\boldsymbol{f}_i|y_{\boldsymbol{b}}=0,I)}\frac{P(y_{\boldsymbol{b}}=1|I)}{P(y_{\boldsymbol{b}}=0|I)} = \prod_{i=1}^{m}\frac{P(y_{\boldsymbol{b}}=1|\boldsymbol{f}_i,I)}{P(y_{\boldsymbol{b}}=0|\boldsymbol{f}_i,I)}. \tag{2}$$

[D] FOD’s detection score after multiple fixations

Derivation for Equation (6):

$$\log\left(\prod_{i=1}^{m}\frac{P(y_{\boldsymbol{b}}=1|\boldsymbol{f}_i,I)}{P(y_{\boldsymbol{b}}=0|\boldsymbol{f}_i,I)}\right)=\sum_{i=1}^{m}\log\left(\frac{P(y_{\boldsymbol{b}}=1|\boldsymbol{f}_i,I)}{1-P(y_{\boldsymbol{b}}=1|\boldsymbol{f}_i,I)}\right) \tag{3}$$

using (5), we get

$$\sum_{i=1}^{m}\log\left(\frac{P(y_{\boldsymbol{b}}=1|\boldsymbol{f}_i,I)}{1-P(y_{\boldsymbol{b}}=1|\boldsymbol{f}_i,I)}\right)=\sum_{i=1}^{m}\log\left(\frac{\frac{1}{1+e^{-s(I,\boldsymbol{b},\boldsymbol{f}_i)}}}{1-\frac{1}{1+e^{-s(I,\boldsymbol{b},\boldsymbol{f}_i)}}}\right) \tag{4}$$

$$=\sum_{i=1}^{m}\log\left(\frac{1}{e^{-s(I,\boldsymbol{b},\boldsymbol{f}_i)}}\right)=\sum_{i=1}^{m}s(I,\boldsymbol{b},\boldsymbol{f}_i). \tag{5}$$

[E] Foveated saliency model

The input image is filtered with a Gabor filter bank having 4 scales (2, 4, 8 and 16 pixels/cycle) and 8 orientations (between 0 and 180 degrees with 22.5-degree increments).

A cell pools (sums) Gabor responses per orientation, within its receptive field.

Let C_θ represent the Gabor magnitude at orientation θ for cell C. The saliency of this cell (i.e. the saliency at the location corresponding to this cell’s receptive field) at orientation θ, $S_{C,\theta}$, is computed as

$$S_{C,\theta}=C_\theta-\frac{1}{n}\sum_{i=1}^{n}D_\theta^{(i)} \tag{6}$$

where $D^{(i)}$ is a neighbor cell of C, the first term is the center response and the second term is the surround response.

The final saliency at cell C is computed as

$$S_C=\arg\max_{\theta}\ S_{C,\theta}. \tag{7}$$

For a foveal cell, we used 8 nearest neighbor cells to compute the surround response. For a peripheral cell, we used the nearest 4 neighboring cells to do the same. In Fig 2, 4 neighbor cells are shown for a peripheral cell on the visual field.

[F] Effect of inhibition-of-return radius on performance

The implementation of inhibition of return (IOR) [5] in the FOD is intended to

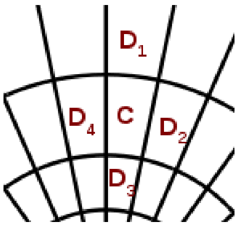

Fig 2. 4 neighbor cells (D1 to D4) are shown on the visual field for the peripheral cell C. The center response is computed at cell C and the surround response is computed using the responses of the 4 neighboring cells.

encourage the model to explore scene through eye movements. We chose a value of 2 degrees to cover the fovea of the FOD. We emphasize that this parameter was selected as a practical choice for our implementation of the FOV and is much larger than the IOR in humans. The dissociation comes about from our implementation of the FOD. To reduce computational complexity of the FOD, within the fovea, all processing is at the same spatial resolution. Thus, a target object results in the same detection scores irrespective of it position within the FOD's fovea. For the FOD, there is no accuracy increase for fixations within the fovea. For humans small saccades have multiple functions: 1. Human saccade endpoints can be biased and undershoot the desired endpoint. Small saccades for humans can serve as corrective. 2. Humans are also limited by internal noise that limits from instant to instant the fidelity of the processed visual information. Thus, for humans a second chance to re-evaluate the adjacent regions of previous fixations would potentially increase the accuracy of detection. However, our model does not include either these motor noise or biases on eye movements and also there is no internal noise that perturbs the acquired signals dynamically. Thus, moving the fovea a small distance along the image will result in the same acquired information (detection score) for the model and little added benefit to accuracy. Our prediction is supported by simulations (Fig 3) varying the area for inhibition of return which show that as we decrease the visual angle parameter controlling the IOR, the average precision recall curve either remains unchanged or diminishes by a small degree. Fig 3 shows the FOD's detection accuracy (for 5 fixations) as a function of the IOR radius for 4 different object classes.

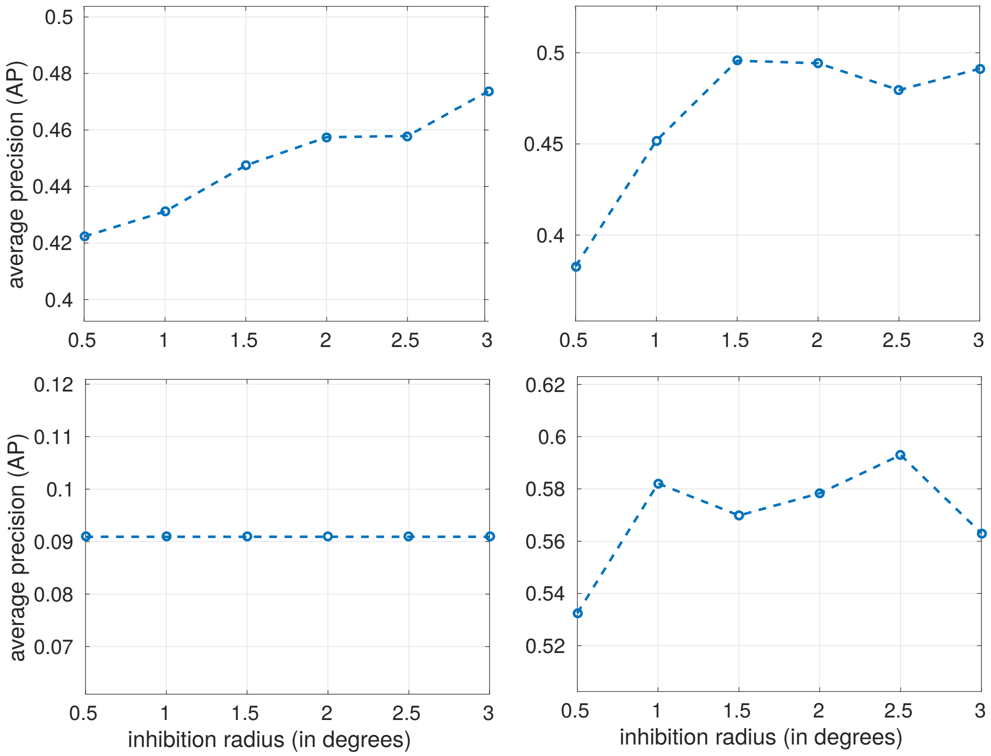

Fig 3. Average precision (AP) achieved by the FOD (using 5 fixations) as a function of different inhibition of return radii for the “bicycle” class (top-left), the “car” class (top-right), the “cow” class (bottom-left), and the “horse” (bottom-right) class.

References

1. Felzenszwalb PF, Girshick RB, McAllester D, Ramanan D. Object Detection with Discriminatively Trained Part Based Models. IEEE Transactions on Pattern Analysis and Machine Intelligence. 2010;32(9):1627–1645.

2. Malisiewicz T, Gupta A, Efros AA. Ensemble of Exemplar-SVMs for Object Detection and Beyond. In: ICCV; 2011.

3. Hariharan B, Malik J, Ramanan D. Discriminative Decorrelation for Clustering and Classification. In: European Conference on Computer Vision; 2012.

4. Girshick RB, Felzenszwalb PF, McAllester D. Discriminatively Trained Deformable Part Models, Release 5;. http://people.cs.uchicago.edu/ rbg/latent-release5/.

5. Ro T, Pratt J, Rafal RD. Inhibition of return in saccadic eye movements. Experimental Brain Research. 2000;130(2):264–268.
